# Biosynthesis of 3-thia-α-amino acids on a carrier peptide

**DOI:** 10.1073/pnas.2205285119

**Published:** 2022-07-05

**Authors:** Yue Yu, Wilfred A. van der Donk

**Affiliations:** ^a^Department of Chemistry, University of Illinois at Urbana–Champaign, Urbana, IL 61801;; ^b^HHMI, University of Illinois at Urbana–Champaign, Urbana, IL 61801

**Keywords:** pearlin, RiPP, radical SAM methyltransferase, carrier protein, 3-thiahomoleucine

## Abstract

A subset of natural products, such as polyketides and nonribosomal peptides, is biosynthesized while tethered to a carrier peptide via a thioester linkage. Recently, we reported that the biosyntheses of 3-thiaglutamate and ammosamide, single amino acid–derived natural products, employ a very different type of carrier peptide to which the biosynthetic intermediates are bound via an amide linkage. During their biosyntheses, a peptide aminoacyl-transfer ribonucleic acid (tRNA) ligase (PEARL) first loads an amino acid to the C terminus of the carrier peptide for subsequent modification by other enzymes. Proteolytic removal of the modified C-terminal amino acid yields the mature product. We termed natural products that are biosynthesized using such pathways pearlins. To investigate the diversity of pearlins, in this study we experimentally characterized another PEARL-encoding biosynthetic gene cluster (BGC) from *Tistrella mobilis* (*tmo*). The enzymes encoded in the *tmo* BGC transformed cysteine into 3-thiahomoleucine both in vitro and in *Escherichia coli*. During this process, a cobalamin-dependent radical *S-*adenosylmethionine (SAM) enzyme catalyzes *C*-isopropylation. This work illustrates that the biosynthesis of amino acid–derived natural products on a carrier peptide is a widespread strategy in nature and expands the spectrum of thiahemiaminal analogs of amino acids that may serve a broader, currently unknown function.

## Significance

Natural products have played an important role in the development of human medicine. A more complete understanding of natural product biosynthesis is important for two directions of high contemporary interest: computer-aided structure prediction of natural products encoded in the genomes and bioengineering of their structures for human applications. Pearlins, amino acid–derived natural products, are biosynthesized at the C terminus of a carrier peptide by the appendage of amino acids and subsequent modifications. This study reports 3-thiahomoleucine as a member of the pearlin natural products. A cobalamin-dependent radical *S-*adenosylmethionine enzyme TmoD performs three consecutive radical methyl transfers during 3-thiahomoleucine biosynthesis. This study shows the diversity of pearlin natural products and expands the range of chemical transformations involved in their biosyntheses.

**Fig. 1.**
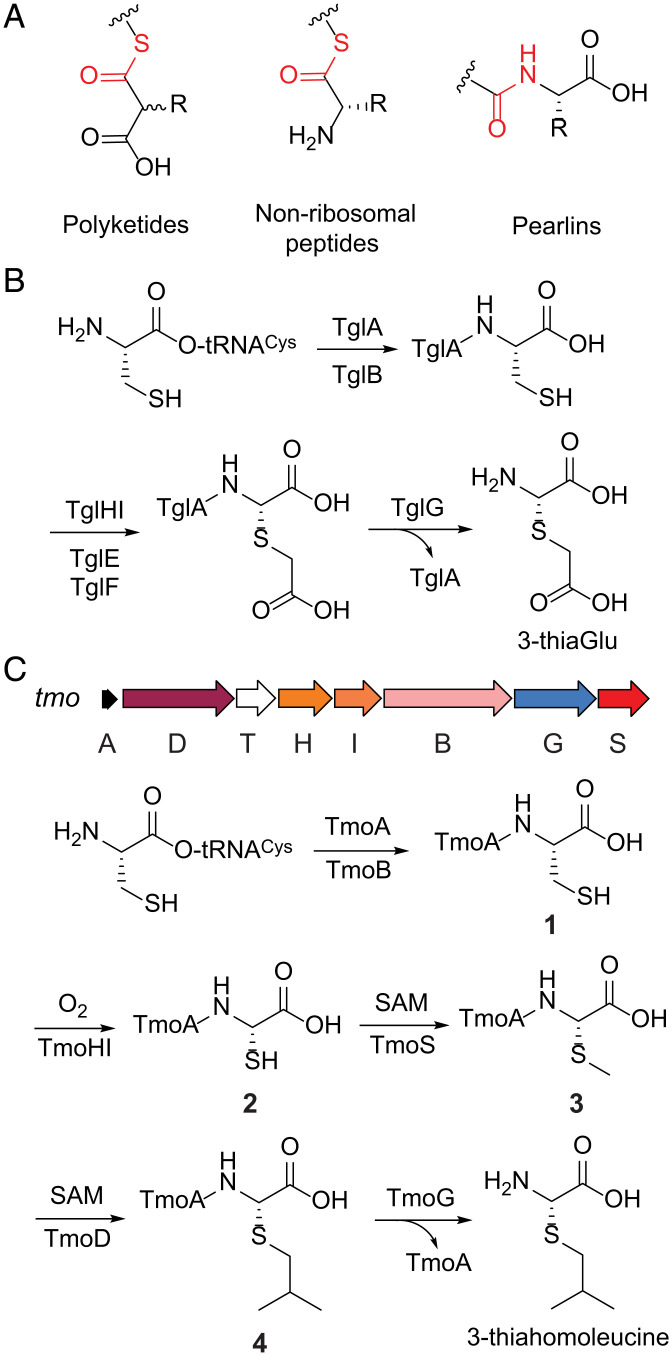
Biosynthesis of natural products on a carrier peptide. (*A*) During polyketide and NRP biosynthesis, the biosynthetic building blocks and intermediates are linked to a carrier protein via a thioester bond. During pearlin biosynthesis, an amino acid is linked to a carrier peptide via an amide bond. (*B*) The biosynthetic pathway of 3-thiaGlu. TglA is the carrier peptide. (*C*) The *tmo* BGC and the reconstituted biosynthetic pathway of 3-thiahomoleucine. The stereochemistry of the final product is based on inference from the 3-thiaGlu pathway.

Biosynthesis of natural products on a carrier peptide is a conserved biosynthetic strategy in nature, encompassing fatty acid, polyketide, and nonribosomal peptide (NRP) biosynthesis (1, 2). The biosynthetic building blocks, such as malonate derivatives (3) and amino acids, are activated and loaded onto the phosphopantetheine arm of a carrier protein via a thioester linkage (Fig. 1*A*). This thioester linkage serves to activate each biosynthetic unit toward iterative chain extension by nucleophilic attack. After chain extension is completed, thioesterases unload the biosynthetic intermediate from the carrier protein to release the mature product. This covalent assembly line strategy is central to the biosynthesis of many natural products of medical importance.

Recently, we discovered that the biosynthesis of amino acid–derived natural products can also employ a different type of carrier peptide (4, 5). In this case, a proteinogenic amino acid is first loaded onto the C terminus of a carrier peptide by a peptide aminoacyl-tRNA ligase (PEARL) (Fig. 1*B*). PEARLs activate the C-terminal carboxylate of the carrier peptide by phosphorylation and append an amino acid by conjugating an aminoacyl-tRNA followed by the hydrolytic removal of the tRNA (6). Other enzymes in the biosynthetic gene cluster (BGC) then utilize the carrier peptide for recognition to further modify the C-terminal amino acid. Finally, a protease releases the modified amino acid to yield the mature product, a class of molecules termed the pearlins (7), and regenerates the carrier peptide. The functions of the PEARL enzyme and the protease during pearlin biosynthesis resemble those of the adenylation domain and the thioesterase during NRP biosynthesis, respectively, although the chemistry of the steps involved is different.

The biosynthesis of amino acid–derived natural products at the C terminus of a carrier peptide was first discovered for 3-thiaglutamate (3-thiaGlu) biosynthesized by enzymes encoded in the *tgl* BGC from the plant pathogen *Pseudomonas syringae* (4). During 3-thiaGlu biosynthesis, cysteine is first appended to the C terminus of the carrier peptide TglA. The appended cysteine undergoes excision of its β-carbon, *S*-carboxymethylation, and proteolytic cleavage to yield 3-thiaGlu (Fig. 1*B*). Similar biosynthetic logic was also demonstrated in the biosynthesis of ammosamides (4, 5, 8), during which a tryptophan is appended to a carrier peptide by a PEARL and other PEARLs utilize glycyl-tRNA as nitrogen donors during further modifications (5); 3-ThiaGlu is an unstable amino acid analog, and its function for the plant pathogen is hypothesized to involve mimicry of glutamate, a signaling molecule in plant defense (9, 10). These two examples raise the question as to how diverse the pearlin structures may be. A survey of the available sequenced genomes suggests that a similar biosynthetic strategy could generate diverse amino acid analogs that, like 3-thiaGlu, would contain a thiahemiaminal, hinting that this structural motif may be more general. Therefore, in this work, we investigated another PEARL-containing BGC (denoted *tmo*) from *Tistrella mobilis*. Bioinformatic analysis of the enzymes encoded in the *tmo* BGC suggests that the early steps in the pathway resemble those of 3-thiaGlu biosynthesis, but differences between the *tmo* and *tgl* BGCs imply a different final product.

In this study, we reconstituted the activity of the enzymes in the *tmo* BGC and show that they generate 3-thiahomoleucine (Fig. 1*C*). Its biosynthesis starts from cysteine addition and excision of its β-carbon, biosynthetic steps shared with 3-thiaGlu. However, these early steps are followed by *S*-methylation and *C*-isopropylation that divert this biosynthetic intermediate to a different final product. We reconstituted the activity of the cobalamin-dependent radical *S-*adenosylmethionine (rSAM) enzyme TmoD responsible for *C*-isopropylation during 3-thiahomoleucine biosynthesis both in vitro and in *Escherichia coli*. Mechanistic studies demonstrate that the *C*-isopropylation occurs by iterative methylation, which involves the generation of the 5′-deoxyadenosyl radical (5′-dA•), hydrogen atom abstraction, and methyl transfer from methylcobalamin. This study illustrates that formation of thiahemiaminal analogs of amino acids is not limited to glutamate and raises the question of the biological function of these structures.

## Results

### TmoB Appends Cysteine to the C Terminus of TmoA.

**Fig. 2.**
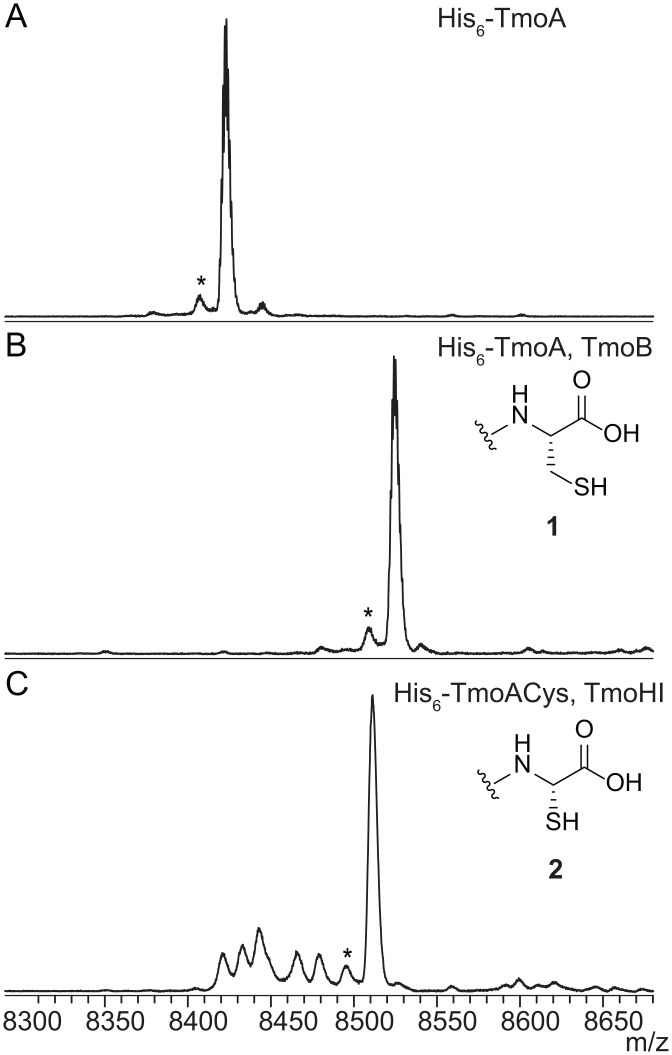
Matrix-assisted laser desorption/ionization time-of-flight mass spectra of TmoA-derived products showing the activity of TmoB and TmoHI in *E. coli*. (*A*) MALDI-TOF MS of the carrier peptide His_6_-TmoA. Average mass-to-charge ratio (m/z) of the singly charged positive ion ([M+H]^+^) calculated: 8,425; observed: 8,423. (*B*) MALDI-TOF MS of His_6_-TmoA coexpressed with TmoB. Average *m/z* [M + H]^+^ for His_6_-TmoACys calculated: 8,528; observed: 8,525. (*C*) TmoHI excised the β-carbon of the C-terminal cysteine. Average *m/z* [M + H]^+^ for **2** calculated: 8,514; observed: 8,511. *These peaks are deamination artifacts observed at high *m/z* values in MALDI-TOF MS.

The genome of the marine α-proteobacteria *T. mobilis* KA081020-065 (11) contains a BGC that encodes a putative carrier peptide (TmoA), a PEARL (TmoB), a DUF692 protein (4, 12) (TmoH), a protein containing a recognition element for ribosomally synthesized and post-translationally modified peptides (RiPPs) (13) (TmoI), a methyltransferase (TmoS), a cobalamin (B_12_)-dependent rSAM enzyme (TmoD), a metalloprotease (TmoG), and a transporter (TmoT) (Fig. 1*C*). Based on the biosynthetic logic of pearlins, an amino acid would be added to the C terminus of the carrier peptide TmoA by the PEARL TmoB. At present, the amino acid specificity of PEARLs cannot yet be predicted (5). TmoB shares 33.8% sequence identity with the cysteine-adding PEARL TglB; 29.1% identity with the alanine-adding BhaB_1_; 22.0 and 41.7% identity with the tryptophan-adding PEARLs BhaB_7_ and AmmB_2_, respectively; and 20.8 and 31.4% identity with the glycine-adding PEARLs BhaB_5_ and AmmB_3_, respectively. To obtain insight into the amino acid specificity of TmoB, we first coexpressed the enzyme with His_6_-TmoA in *E. coli*. After immobilized metal affinity chromatography (IMAC) purification, matrix-assisted laser desorption/ionization time-of-flight mass spectrometry (MALDI-TOF MS) analysis demonstrated the production of peptide **1** (Fig. 2 *A* and *B*). The mass difference of this product with His_6_-TmoA is consistent with the addition of cysteine. This conclusion was supported by iodoacetamide (IAA) labeling (*SI Appendix*, Fig. S1) and electrospray ionization high-resolution tandem mass spectrometry (ESI-HRMS/MS) analysis (*SI Appendix*, Figs. S2 and S3). The observed cysteine addition makes TmoB the second cysteine-adding PEARL besides TglB, despite sharing only 33.8% sequence identity.

### TmoHI Excises the β-Carbon of the Added Cysteine.

Since TmoB performed cysteine addition similar to TglB from 3-thiaGlu biosynthesis, we hypothesized that TmoH and TmoI (hereafter referred to as TmoHI) would cooperate to excise the β-carbon of the cysteine, akin to the activity of TglHI from the *tgl* BGC (4). By coexpressing His_6_-TmoACys (cysteine genetically encoded at the C terminus) and TmoHI, a product with a mass change of −14 Da was observed by MALDI-TOF MS (Fig. 2*C*). Labeling with IAA supported the expectation that the thiol group was retained during this transformation (*SI Appendix*, Fig. S4), and ESI-HRMS/MS of this coexpression product was consistent with the assignment of structure **2** as the product of TmoHI (Fig. 1*C* and *SI Appendix*, Fig. S5).

### TmoS Methylates the Thiol of Peptide 2.

**Fig. 3.**
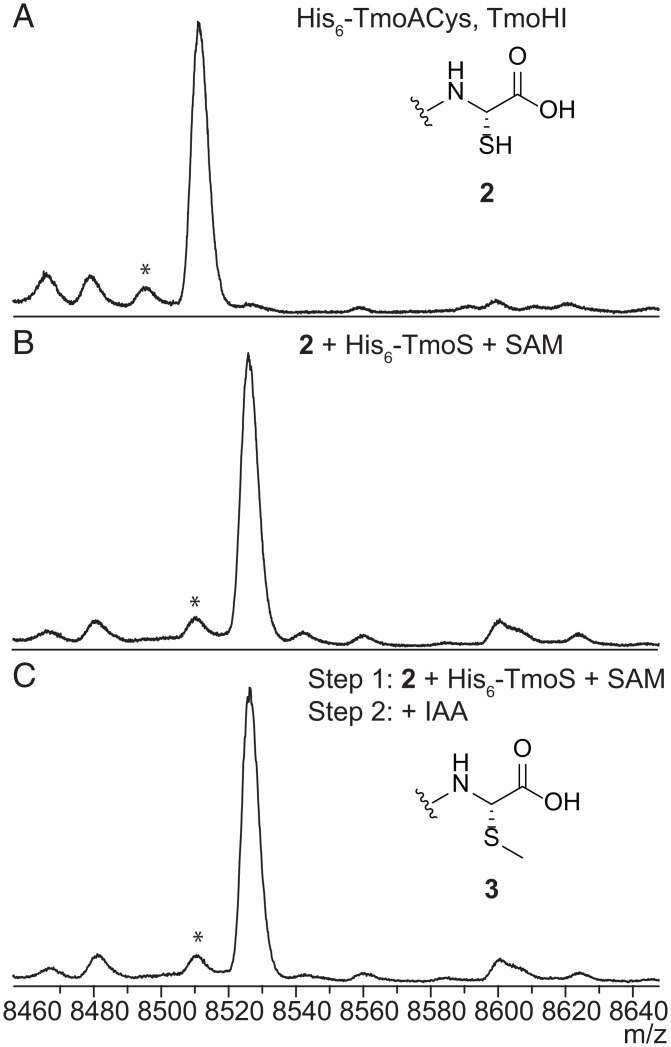
Matrix-assisted laser desorption/ionization time-of-flight mass spectra demonstrating that TmoS methylates **2** both in vitro and in *E. coli*. (*A*) Peptide **2** generated by coexpressing His_6_-TmoACys and TmoHI in *E. coli*. Average *m/z* [M + H]^+^ calculated: 8,514; observed: 8,511. (*B*) Reaction of **2** with His_6_-TmoS and SAM. Average *m/z* [M + H]^+^ calculated: 8,528; observed: 8,526. (*C*) Treatment of the reaction product shown in *B* with IAA. Average *m/z* [M + H]^+^ calculated: 8,528; observed: 8,526. *These peaks are deamination artifacts observed at high *m/z* values in MALDI-TOF MS.

While the TmoB- and TmoHI-mediated transformations to install a C-terminal 2-mercaptoglycine are similar to 3-thiaGlu biosynthesis in *P. syringae* (4), the presence of two genes encoding different modifying enzymes, TmoS and TmoD, suggests that the product of the *tmo* BGC would be different from 3-thiaGlu. We separately purified **2** and recombinant His_6_-TmoS after heterologous production in *E. coli*. Analysis of an in vitro reaction using **2**, His_6_-TmoS, and *S-*adenosylmethionine (SAM) by MALDI-TOF MS demonstrated conversion of peptide **2** to a +14-Da product, consistent with methylation (Fig. 3 *A* and *B*). Unlike **2**, this product did not react with IAA (Fig. 3*C*), suggesting that the thiol group of **2** was methylated to produce **3**. The same product was also obtained by coexpressing His_6_-TmoACys, TmoHI, and TmoS in *E. coli*. Although **3** cannot be distinguished from His_6_-TmoACys by mass, the coexpression product did not react with IAA, suggesting the complete modification of His_6_-TmoACys to **3** (*SI Appendix*, Fig. S6). The transformation was also supported by ESI-HRMS/MS (*SI Appendix*, Fig. S7), as peptide **3** showed distinct fragmentation patterns from a TmoACys standard. TmoS did not methylate His_6_-TmoACys during the same reaction period in vitro (*SI Appendix*, Fig. S6), suggesting that TmoS selectively recognizes peptide **2** for its activity.

### TmoD Installs an Isopropyl Group on Peptide 3.

**Fig. 4.**
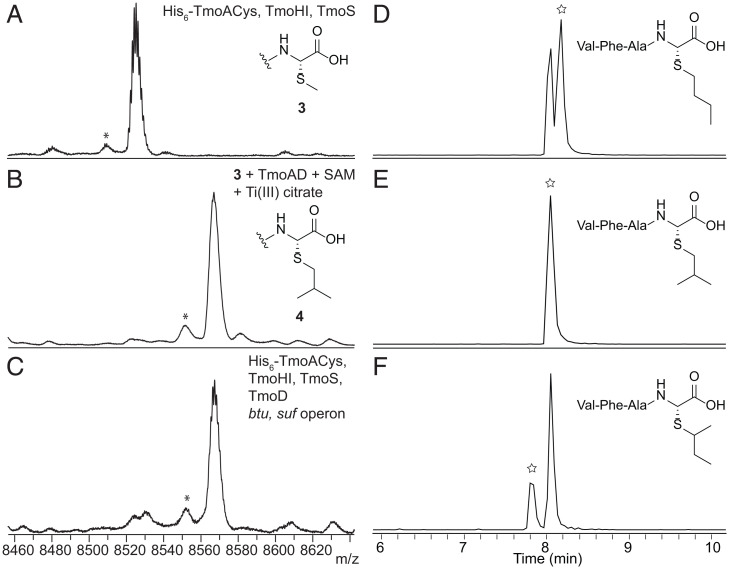
Matrix-assisted laser desorption/ionization time-of-flight mass spectra showing the trimethylation of peptide **3** by TmoD in vitro and in *E. coli* and EICs (*m/z* 481.2479) of trypsin-treated peptide **4** coinjected with different VFAX peptide standards. (*A*) Peptide **3** produced in *E. coli* by coexpressing His_6_-TmoACys, TmoHI, and TmoS. Average *m/z* [M + H]^+^ calculated: 8,528; observed: 8,525. (*B*) Treatment of peptide **3** with reconstituted TmoAD complex, SAM, and Ti(III) citrate in vitro. Average *m/z* [M + H]^+^ calculated: 8,570; observed: 8,567. (*C*) Product of coexpression of His_6_-TmoACys, TmoHI, TmoS, TmoD, and the *btu* and *suf* operons in *E. coli*. Average *m/z* [M + H]^+^ calculated: 8,570; observed: 8,567. *These peaks are deamination artifacts observed at high *m/z* values in MALDI-TOF MS. (*D*) EIC of the coinjection of the *n*-butyl–containing standard and trypsin-treated peptide **4**. (*E*) Coinjection of the isobutyl-containing standard and trypsin-treated peptide **4**. (*F*) Coinjection of the *sec*-butyl–containing standard and the trypsin digest of peptide **4**. The stars denote the standards drawn in each panel.

The remaining modifying enzyme of the *tmo* BGC, TmoD, is bioinformatically predicted to be a class B vitamin B_12_–dependent rSAM enzyme (14). This enzyme family canonically uses SAM, methylcobalamin, and a [4Fe-4S]^2+^ cluster to initiate methyl transfer onto nonnucleophilic phosphorous or carbon atoms (15). Only a few B_12_-dependent rSAM methylation enzymes have been characterized in vitro (16–24). TmoD was expressed aerobically in *E. coli* as a recombinant His_6_-fusion protein that did not contain cobalamin and a [4Fe-4S]^2+^ cluster. This protein did not modify **3** in vitro after anaerobic reconstitution with Na_2_S, (NH_4_)_2_Fe(SO_4_)_2_, and hydroxocobalamin (HOCbl). Serendipitously, we discovered that the apo-TmoD copurified with His_6_-TmoA during IMAC when the two proteins were coexpressed in *E. coli*. Native ESI-MS analysis supported complex formation between His_6_-TmoA and TmoD (*SI Appendix*, Fig. S8). This complex of TmoA and TmoD (the TmoAD complex), after anaerobic reconstitution with Na_2_S, (NH_4_)_2_Fe(SO_4_)_2_, and HOCbl, trimethylated peptide **3** in vitro in the presence of SAM to yield peptide **4**, as demonstrated by a +42-Da mass shift (Fig. 4 *A* and *B*). The reconstituted TmoAD complex contained 4.2 ± 0.9 Fe, 7.0 ± 2.7 sulfide, and 0.93 ± 0.11 HOCbl per protein. The C-terminal 37 amino acids of the TmoA peptide (His_6_-TmoA_37mer_Cys) were sufficient for enzymatic modification by TmoHI, TmoS, and TmoD (*SI Appendix*, Figs. S9 and S10). We screened different in vitro reduction systems, including Ti(III) citrate; methyl viologen with NADPH; and a combination of *E. coli* flavodoxin, flavodoxin reductase, and NADPH, to support the activity of the TmoAD complex. Ti(III) citrate proved to be the most effective to achieve trimethylation (*SI Appendix*, Fig. S10). ESI-HRMS/MS data demonstrated that the trimethylation occurred on the last residue of peptide **3** (*SI Appendix*, Fig. S11). The same product was also obtained by coexpressing His_6_-TmoACys, TmoHI, TmoS, and TmoD in *E. coli* in the presence of the B_12_ uptake plasmid pBADCDF-btu (22, 25) and the [4Fe-4S]^2+^ incorporation plasmid pACYC-sufABCDSE (26) (Fig. 4*C*). Therefore, the same activity of TmoD was observed in vitro and in *E. coli*.

To investigate whether TmoD could modify peptide **2**, we coexpressed His_6_-TmoACys, TmoHI, and TmoD in the presence of pBADCDF-btu and pACYC-sufABCDSE. No further modification beyond the expected activity of TmoHI was observed (*SI Appendix*, Fig. S12). These results collectively showed that the reaction catalyzed by TmoD followed that of TmoS.

We hypothesized that the trimethylation occurred iteratively on the methyl group of the thioether in peptide **3** and investigated the structure of the product using liquid chromatography–electrospray ionization-high-resolution mass spectrometry (LC-ESI-HRMS) coinjection assays. After trimethylation of peptide **3**, the alkyl chain on the sulfur atom could be *n*-butyl, isobutyl, *sec*-butyl, or *tert*-butyl. Therefore, we prepared standards by reacting **2** with *n*-butyl bromide, isobutyl bromide, or *sec*-butyl iodide. The standards and the product of TmoD were digested by trypsin to produce the C-terminal 4-mer peptides valine-phenylalanine-alanine-X (VFAX; X denoting the cysteine-derived amino acid). Chromatography conditions that gave separation of the three standards were identified (*SI Appendix*, Fig. S13), and the product of TmoD was coinjected with each standard. The extracted ion chromatograms (EICs) showed that the VFAX peptide with the isobutyl group coeluted with the product of TmoD, whereas the other standards did not (Fig. 4 *D–F*). We ruled out the *tert*-butyl group–containing VFAX peptide as the product of TmoD by the deuterium-labeling experiments discussed below. These findings support the structure of peptide **4** as having an isobutyl chain connected to the sulfur atom (Fig. 4*B*). Other B_12_-dependent rSAM enzymes that methylate their substrates consecutively in vitro include CysS (20, 27), ThnK (18), and TokK (24, 28) (*SI Appendix*, Fig. S14). CysS methylates the methoxy group of its substrate up to three times to generate a *tert*-butoxy group, and ThnK installs the C6-ethyl chain of thienamycin. The reaction catalyzed by TokK is most similar to TmoD in that this enzyme installs the C6-isopropyl chain of the carbapenem asparenomycin. TmoD is present as a singleton in a recently published sequence similarity network for cobalamin-dependent rSAM enzymes (29, 30), whereas CysS, ThnK, and TokK all fall in the same group of enzymes (*SI Appendix*, Fig. S15). The trimethylation by TmoD to form an isopropyl group on a peptide-bound methylthioether further expands the reaction diversity of B_12_-dependent rSAM methylation enzymes.

### Catalytic Mechanism of TmoD.

**Fig. 5.**
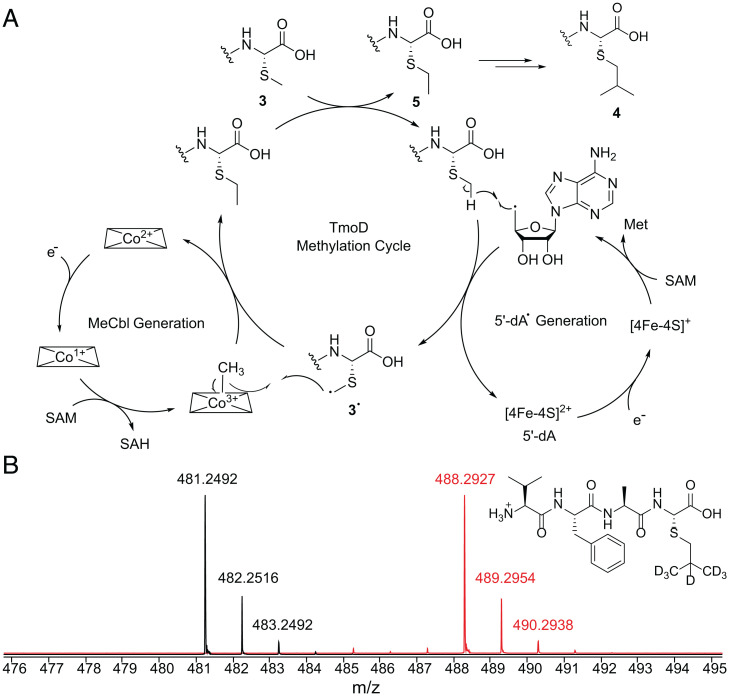
Mechanistic proposal for TmoD catalysis. (*A*) Proposed catalytic cycle of TmoD. Only the first methylation step is illustrated in detail. The second and third methylation steps would follow similar mechanisms. (*B*) LC-ESI-HRMS of the trimethylated product when SAM (black) or d_3_-SAM (red) was used. Unlabeled product *m/z* [M + H]^+^ calculated: 481.2479; observed: 481.2492 (2.70-ppm error). d_7_-product *m/z* [M + H]^+^ calculated: 488.2919; observed, 488.2927 (1.64-ppm error). SAH, *S*-adenosyl homocysteine.

Given the structure of peptide **4** and recent studies on TokK (24, 28), we propose the following reaction mechanism for the in vitro reaction catalyzed by TmoD (Fig. 5*A*). The HOCbl is reduced to cob(I)alamin, which reacts with SAM to produce methyl cob(III)alamin. The [4Fe-4S]^2+^ cluster is reduced by the external reductant, and the [4Fe-4S]^+^ cluster initiates production of the 5′-dA• from a second molecule of SAM, possibly with the intermediacy of an organometallic intermediate Ω (31, 32). The 5′-dA• abstracts a hydrogen atom from the methylthio group of peptide **3** to produce **3**•. The methylcobalamin then homolytically transfers the methyl group to **3**• to produce the monomethylated product **5**. Such methyl transfer will occur another two times on this newly added methyl group. Different methylation states were observed during the in vitro process, supporting a sequential, nonprocessive methylation mechanism (*SI Appendix*, Fig. S10), which is also observed for TokK (24). According to this mechanistic proposal, use of d_3_-SAM as the cosubstrate for TmoD in vitro would result in d_7_-**4**, 5′-deoxyadenosine (5′-dA), and d_1_-5′-dA. The maximal percentage of d_1_-5′-dA formed is 67% because the hydrogen atom abstraction in the first methylation step occurs on the unlabeled methylthioether of peptide **3**; the hydrogen atom abstractions in the last two methylation steps would take place from the d_3_-methyl groups appended from d_3_-SAM during the first methylation step. LC-ESI-HRMS analysis showed the incorporation of seven deuterium atoms in peptide **4** (Fig. 5*B*) and production of 58% d_1_-5′-dA (*SI Appendix*, Fig. S16). The lower deuterium incorporation in 5′-dA than predicted could result from an incomplete reaction (mono- or dimethylation) or from uncoupled SAM cleavage (33, 34). These findings also rule out a *sec*-butyl or *tert*-butyl group in the side chain of the C-terminal residue, as d_8_- or d_9_-**4** would have been produced, respectively (*SI Appendix*, Fig. S17). d_3_-Methylcobalamin was formed in these assays from HOCbl, consistent with its role as an intermediate methyl carrier (*SI Appendix*, Fig. S16). To corroborate this mechanistic proposal, we also prepared the putative monomethylated intermediate d_5_-**5** by reacting **2** with d_5_-EtI and subjected the peptide to dimethylation by the TmoAD complex in vitro. The mass shift of the product was consistent with the loss of two deuterium atoms in the dimethylation product (*SI Appendix*, Fig. S18). This experiment further argues against the presence of an *n*-butyl group in **4**, as loss of only one deuterium would have been observed. Collectively, the results from the isotope-labeling experiments and the LC-ESI-HRMS coinjection assays show that TmoD installed an isopropyl group on the methyl thioether of peptide **3**.

### TmoG Cleaves off the C-Terminal 3-Thiahomoleucine.

Based on the biosynthetic logic of the previously studied pearlins (4, 5), the membrane-bound protease TmoG was anticipated to cleave the C-terminal 3-thiahomoleucine from peptide **4** and regenerate the scaffold peptide His_6_-TmoA. Therefore, we treated **4** with the cell lysate of *E. coli* expressing His_6_-TmoG and with the lysate of *E. coli* containing an empty plasmid as control. The C-terminal 3-thiahomoleucine on peptide **4** was removed by the His_6_-TmoG–containing lysate but not the control (*SI Appendix*, Fig. S19). We also explored the substrate preference of TmoG by testing whether related peptides (peptide **3**, TmoA-Cys, TmoA-Leu, TmoA-Lys, TmoA-Gln, and TmoA-Met) would be processed. Among the peptides evaluated, only TmoA-Met was significantly processed (*SI Appendix*, Fig. S19). The substrate preference of TmoG is consistent with the hydrophobic and extended side chain of peptide **4**. The thiahemiaminal moiety in 3-thiahomoleucine (3-thiahLeu) is susceptible to hydrolysis, and its short lifetime renders MS detection difficult. However, despite the unstable nature of 3-thiahLeu, all evidence points at another example of a BGC that produces a thiahemiaminal analog of an amino acid.

## Discussion

In this study, we reconstituted the biosynthetic enzymes of the *tmo* BGC from *T. mobilis* and showed they produce 3-thiahLeu on the TmoA scaffold peptide. After TmoB appends Cys to the C terminus of TmoA, TmoHI catalyzes β-carbon excision, TmoS promotes *S*-methylation, and TmoD introduces *C*-isopropylation. Finally, TmoG removes the modified amino acid at the C terminus to yield 3-thiahLeu. Although the function of this unstable product is currently unknown, the structures of 3-thiaGlu and 3-thiahLeu may provide insights into the evolutionary origin of using an amide linkage to a scaffold peptide rather than the ubiquitous thioester linkage in NRP biosynthesis. The amide linkage during the biosynthesis of 3-thia amino acids prevents the hydrolysis of the biosynthetic intermediates that would have occurred if they were linked through their carboxylates. The BGC of 3-thiaGlu is found in the plant pathogen *P. syringae*, and we have previously suggested that 3-thiaGlu might mimic glutamate to act on plant glutamate receptors, possibly covalently. The purpose of synthesizing 3-thiahLeu by *T. mobilis* is not clear. BGCs homologous to *tmo* are found not only in α-proteobacteria but also in actinobacteria (*SI Appendix*, Fig. S20). They are likely to also biosynthesize 3-thiahLeu or close analogs.

One possibility that we cannot rule out is that the peptides containing the thiahemiaminal group are the actual desired products in the biosynthetic pathways discussed herein. Such peptides could, for instance, inactivate carboxypeptidases or proteases by potentially releasing a reactive electrophile upon attack at the C-terminal amide carbonyl. In this model, the proteases encoded in the BGC that remove the C-terminal structures would be self-immunity proteins. Arguing against such a model is that the BGCs of 3-thiaGlu and 3-thiahLeu do not contain any peptide transporters, but these may be located elsewhere in the genome as observed for other RiPPs (35). Regardless of their biological function, the current study demonstrates that 3-thia-α-amino acids extend beyond 3-thiaGlu and appear to be generated in several bacterial phyla.

**Fig. 6.**
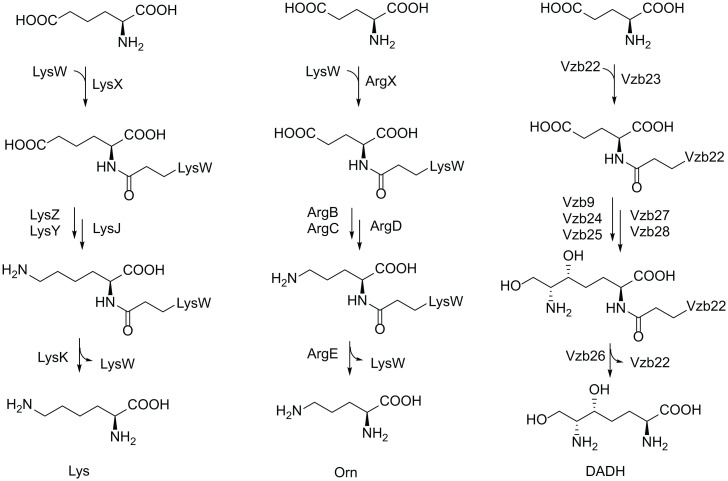
AmCP-mediated biosynthesis of lysine (Lys), ornithine (Orn), and DADH. LysW and Vzb22 are the AmCPs.

The pearlin biosynthetic pathways have several commonalities with another scaffold-assisted route to amino acid analogs. Initially discovered during investigations of lysine biosynthesis in *Thermus thermophilus* (36), amino group carrier proteins (AmCPs) use activation of the γ-carboxylate of a C-terminal glutamate residue to form an isopeptide bond to the amino group of various cellular building blocks (Fig. 6). Subsequent modifications to that building block result in the formation of the final product bound via the isopeptide linkage to the AmCP, followed by hydrolytic liberation of the final product. This biosynthetic strategy is used for the generation of proteinogenic amino acids lysine and arginine (37) and the nonproteinogenic amino acid (2*S*,6*R*)-diamino-(5*R*,7)-dihydroxy-heptanoic acid (DADH) (Fig. 6), which is involved in the production of NRP natural products, such as vazabitide A (38) and s56-p1 (39). It is possible that the 3-thia-α-amino acids are also incorporated into other natural product biosynthetic pathways, like a PEARL-derived pyrroloiminoquinoline that is used by a polyketide synthase during lymphostin biosynthesis (40). The AmCPs are thought to prevent unfavorable off-pathway reactions of intermediates and to assist substrate recognition by the biosynthetic enzymes. These two roles of the AmCPs are also fulfilled by the scaffold proteins in the biosynthesis of 3-thia-α-amino acids.

In addition to these similarities, the carrier peptides employed in the biosynthesis of pearlins also have several features that are different from the AmCPs. The N-terminal globular domain and the C-terminal extension of the AmCPs are both used for recognition by their modifying enzymes (37, 41). In contrast, the N-terminal sequences of the carrier protein employed in the biosynthesis of 3-thiaGlu (6, 42) and 3-thiahLeu are dispensable, indicating that their biosynthetic enzymes recognize only the C-terminal sequences of the carrier protein. Each modifying enzyme in the BGC of 3-thia-α-amino acids appears to recognize different sections of the carrier protein since the minimum sequence requirements of various modifying enzymes are different (6, 42). In addition, enzymes acting on AmCP-tethered substrates use electrostatic interactions between positively charged residues on the enzyme and negatively charged residues on the AmCPs (37, 41). In the case of 3-thia-α-amino acids, mutational studies have shown that both electrostatic interactions and hydrophobic interactions are used in the recognition of the carrier proteins (6, 42). The conserved phenylalanine-X (any amino acid)-leucine-aspartate/glutamate (FXLD/E) motif of the carrier protein might bind to the RiPP recognition elements of the modifying enzymes using hydrophobic interactions in ways similar to the antiparallel β-strand that is observed in the class I lanthipeptide biosynthetic systems (43).

In summary, generation of thiahemiaminal mimics of various amino acids on carrier peptides appears to be a common strategy in diverse phyla. Although the biological activity of the products remains to be determined, the pathways to these structures are another example of nature’s propensity for building natural products while covalently tethered to a small scaffold protein.

## Materials and Methods

### Expression, Purification, and Anaerobic Reconstitution of the TmoAD Complex.

*E. coli* codon-optimized *tmoA* was cloned into pRSFDuet-1 multiple cloning site (MCS) I after the sequence encoding a His_6_ tag. *E. coli* codon-optimized *tmoD* was cloned into pRSFDuet-1 MCS II. A 5-mL overnight culture of *E. coli* BL21 (DE3) cells harboring the above plasmid in Luria–Bertani (LB) medium supplemented with 50 mg/L kanamycin was used to inoculate 500 mL of LB medium with 50 mg/L kanamycin in a 2-L baffled shake flask. The culture was grown at 37 °C at 220 rpm until the optical density at 600 nm (OD600) reached 0.6 to 1. The culture was cooled at 4 °C for 1 h, and protein expression was induced with 0.2 mM isopropyl-1-thio-β-D-galactoside. The culture was shaken for another 16 h at 18 °C at 220 rpm. Cells were harvested by centrifugation at 5,000 × *g* for 15 min and resuspended in 25 mL of purification buffer containing 50 mM 4-(2-hydroxyethyl)-1-piperazineethanesulfonic acid (Hepes), 300 mM NaCl, and 10% glycerol, pH 7.6. Cells were lysed on ice by sonication at 40% amplitude for 2 s on and 5 s off for a total time of 15 min. The lysate was clarified by centrifugation at 49,000 × *g* for 15 min. The supernatant was incubated with preequilibrated HisPur cobalt resin (ThermoFisher) for 10 min at 4 °C. The resin was sequentially washed with 5, 10, and 20 mM imidazole in purification buffer. The TmoAD complex was eluted with 200 mM imidazole in purification buffer. The protein was concentrated, and the buffer was exchanged into the purification buffer using a 50-kDa molecular mass cutoff filter (Millipore) to a final volume of around 300 μL. The protein solution was flash frozen and stored at −80 °C until reconstitution. Protein concentration was estimated by *A*_280_ by NanoDrop. Typical yield of protein was 10 mg/L. The frozen protein stock was imported into an anaerobic chamber (Coy). Dithiothreitol (DTT), Na_2_S, (NH_4_)_2_Fe(SO_4_)_2_, HOCbl, and aerobically purified TmoAD stock solutions were sequentially added to degassed purification buffer to achieve a final volume of 1 mL. The final concentrations of each reagent were 3 mM, 500 μM, 500 μM, 500 μM, and 5 mg/mL, respectively. After 8 h of reconstitution on ice, the protein solution was desalted anaerobically using a PD-10 column (GE Biosciences) equilibrated with purification buffer supplied with 3 mM DTT. The eluted protein was brown in color. Residual-free HOCbl was removed anaerobically by three rounds of dilution and concentration using a 50-kDa molecular mass cutoff filter. Protein concentration of the reconstituted TmoAD complex was estimated by the Bradford assay using bovine serum albumin as the standard. Reconstituted protein was aliquoted anaerobically, flash frozen, and stored in a liquid nitrogen Dewar.

### In Vitro Assay of TmoAD Activity.

The in vitro assay was carried out using either full-length peptide **3**, the C-terminal 38-mer of peptide **3**, or full-length peptide **5**. A 50-μL reaction was set up anaerobically in 50 mM Hepes and 100 mM NaCl, pH 7.6, containing 20 to 180 μM substrate, 4 to 20 μM enzyme (the case by case concentrations are specified in *SI Appendix*), and 1 to 2 mM SAM or d_3_-SAM. The reaction was initiated by adding 2 mM Ti(III) citrate (final concentration). The reaction proceeded in the dark for 16 h. A 10-μL aliquot was taken out from the anaerobic chamber for analysis with MALDI-TOF MS after cleanup with a Ziptip C18 (Agilent Technologies). If the reaction conversion was satisfactory, the remainder of the reaction was taken out from the anaerobic chamber, diluted with an equal volume of 50 mM ammonium bicarbonate, and digested with 2 μg of sequencing grade trypsin (Worthington) at 37 °C for 8 h. After the digestion, the reaction was acidified with 1% formic acid and cleaned up with a TopTip C18 column (Glygen). Tryptic fragments were eluted with 60% acetonitrile + 0.1% formic acid. The elution was lyophilized and redissolved in H_2_O for subsequent LC-ESI-HRMS analysis.

## Supplementary Material

Supplementary File

## Data Availability

All study data are included in the article and/or *SI Appendix*.
