# Supplementary material for: Biosynthesis of 3-thia-α-amino acids on a carrier peptide
Source: Proc Natl Acad Sci U S A. 2022 Jul 5;119(29):e2205285119. doi: 10.1073/pnas.2205285119 (PMC9303977; doi:10.1073/pnas.2205285119)
Supplement: Supplementary File [file pnas.2205285119.sapp.pdf]

Supporting Information Appendix

Biosynthesis of 3-thia-α-amino acids on a carrier peptide

Yue Yu and Wilfred A. van der Donk*

Department of Chemistry and Howard Hughes Medical Institute, University of Illinois at Urbana-Champaign, Urbana, IL 61801

Email: vddonk@illinois.edu

General materials and methods

All oligonucleotides used in this study were purchased from Integrated DNA Technologies. Gblocks for *Escherichia coli* (*E. coli*) codon-optimized genes in the *tmo* BGC were purchased from Twist Bioscience. Enzymes and reagents used for molecular biology experiments were purchased from New England BioLabs. For immobilized metal affinity chromatography (IMAC), His60 Ni Superflow Resin was obtained from Takara and HisPur™ Cobalt Resin was procured from Thermo Scientific. Super DHB, 10-15% $TiCl_3$ solution in 12% hydrochloric acid, *n*-butyl bromide, isobutyl bromide, *sec*-butyl iodide, hydroxocobalamin and reduced nicotinamide adenine dinucleotide phosphate (NADPH) were purchased from Sigma-Aldrich. Sequencing grade trypsin was obtained from Worthington Biochemical Corporation. *E. coli* DH5α and DH10β were used for cloning and plasmid maintenance. *E. coli* BL21(DE3) cells were used for protein overexpression. Matrix-assisted laser absorption/desorption ionization time-of-flight mass spectrometry (MALDI-TOF MS) analysis was performed using a Bruker UltrafleXtreme MALDI TOF-TOF mass spectrometer (Bruker Daltonics) at the University of Illinois School of Chemical Sciences Mass Spectrometry Laboratory. MALDI-TOF MS samples were desalted prior to analysis by using a C18 ZipTip (Agilent Technology) prior to co-crystallization in super-DHB as the matrix. Liquid chromatography electrospray ionization high resolution mass spectrometry (LC-ESI-HRMS) was performed using an Agilent 1260 Infinity II HPLC coupled with an Agilent G6545B quadrupole- time-of-flight (qTOF) mass spectrometer. LC-HRMS samples were purified using TopTip2 C18 (Glygen) prior to injection. Native ESI-MS analysis was performed on a Q Exactive UHMR Hybrid Quadrupole-Orbitrap MS system (ThermoFisher Scientific) with an Advion TriVersa NanoMate nanoelectrospray ion source.

Generation of coexpression constructs

Coexpression plasmid for His_6-TmoA and TmoB

E. coli codon-optimized *tmoA* was cloned into pRSFDuet-1 multiple cloning site one (MCSI) by Gibson assembly. The primers TmoAF, TmoAR, TmoA_pRSF_F and TmoA_pRSF_R (Table S1) were used to generate pRSFDuet:His_6-TmoA. *E. coli* codon-optimized *tmoB* was cloned into the MCSII of the above construct to generate pRSFDuet:His_6-TmoA:TmoB. The primers TmoB1F,

TmoB1R, TmoB2F, TmoB2R, TmoB_BBF, and TmoB_BBR (Table S1) were used to generate this construct.

Coexpression plasmid for His_6-TmoACys and TmoHI

The codon encoding for a C-terminal cysteine was introduced into the construct pRSFDuet:His_6-TmoA by site-directed mutagenesis. Primers TmoACysF and TmoACysR (Table S1) were used to generate pRSFDuet:His_6-TmoACys. *E. coli* codon-optimized *tmoHI* was cloned immediately after the coding sequence of His_6-TmoACys with a ribosomal binding site (RBS) spacer (AAGGAGATATACA). The primers TmoHI_F, TmoHI_R, TmoHI_BBF and TmoHI_BBR (Table S1) were used to construct pRSFDuet:His_6-TmoACys_TmoHI: empty.

Coexpression plasmid for His_6-TmoACys, TmoHI and TmoS

E. coli codon-optimized *tmoS* was cloned into the MCSII of pRSFDuet:His_6-TmoACys_TmoHI:empty by Gibson Assembly to generate pRSFDuet:His_6-TmoACys_TmoHI:TmoS. The primers TmoSF, TmoSR, TmoS_BBF and TmoS_BBR (Table S1) were used to make this construct.

Coexpression plasmid for His_6-TmoACys, TmoHI, TmoS and TmoD

E. coli codon-optimized *tmoS* and *tmoD* was cloned into the MCS II of pRSFDuet:His_6-TmoACys_TmoHI:empty by Gibson Assembly to generate pRSFDuet:His_6-TmoACys_TmoHI:TmoS_TmoD. The primers TmoSF, TmoSD_g1R, TmoSD_g2F, TmoD_R, TmoD_BBF and TmoS_BBR (Table S1) were used to make this construct.

Coexpression plasmid for His_6-TmoACys, TmoHI and TmoD

The nucleotide sequence between *tmoHI* and *tmoD* in the construct pRSFDuet: His_6-TmoACys_TmoHI:TmoS_TmoD was deleted using the NEB Q5 site-directed mutagenesis method to generate the plasmid pRSFDuet:His_6-TmoACys_TmoHI_TmoD. The primers TmoACysHID_F and TmoACysHID_R (Table S1) were used to generate this construct.

Coexpression plasmid for His_6-TmoA and TmoD

E. coli codon-optimized *tmoD* was cloned into the MCS II of pRSFDuet:His_6-TmoA:empty by Gibson Assembly to generate pRSFDuet:His_6-TmoA:TmoD. The primers TmoD_F, TmoD_R, TmoD_BBF and TmoD_BBR (Table S1) were used to generate this construct.

Cobalamin (B_{12}) uptake helper plasmid pBADCDF-btu

The cobalamin uptake genes from the construct btu-pBAD1030C-2 (1) were amplified by PCR and inserted into the vector pBADCDF (CDF-based vector with arabinose-inducible protein expression) using Gibson Assembly to yield pBADCDF-btu. The primers btu-pBADCDF_F, btu-pBADCDF_R, pBAD1030_btuF and pBAD1030_btuR (Table S1) were used to generate this construct.

Procedures for coexpressions involving TmoB, TmoHI and TmoS

E. coli BL21(DE3) was transformed with the construct for each coexpression by the KCM method (2) and selected overnight at 37 °C on Luria–Bertani (LB) agar with 50 µg/mL kanamycin. A single colony from the transformation was inoculated into 5 mL of LB with 50 µg/mL kanamycin and grown at 37 °C at 220 rpm overnight. The starter culture was used to inoculate 500 mL of LB with 50 mg/L kanamycin and grown to OD_{600} 0.6-1. The culture was chilled at 4 °C for 1 h and protein expression was induced using 0.2 mM isopropyl-1-thio-β-D-galactoside (IPTG). The culture was incubated for another 16 h at 18 °C at 220 rpm before harvesting.

Procedures for coexpressions involving TmoD

The construct pRSFDuet:His_6-TmoACys_TmoHI:TmoS_TmoD or pRSFDuet:His_6-TmoACys_TmoHI_TmoD was used to transform *E. coli* BL21(DE3) cells containing pBADCDF-btu and pACYC-sufABCDSE , which harbors genes of the *E. coli suf* operon (3). The cells were selected overnight at 37 °C on LB agar with 40 µg/mL kanamycin, 40 µg/mL spectinomycin and 20 µg/mL chloramphenicol. A single colony from the transformation was inoculated into 5 mL of LB with the same concentration of three antibiotics mentioned above and grown at 37 °C at 220 rpm overnight. The starter culture was inoculated into 1 L of LB medium with the same concentration of three antibiotics and grown at 37 °C at 220 rpm until the OD_{600} reached 0.6. Then 2 g (0.2 % *w*/*v*) arabinose, and 2 µM (final concentration) aqueous hydroxocobalamin (HOCbl) were added to the culture. The culture was incubated at 30 °C at 220 rpm for 2 h to allow the genes for B_{12} uptake to express. After that, the culture was chilled at 4 °C for 1 h, and the expression of the genes for $[4Fe\text{-}4S]^{2+}$ cluster assembly and the biosynthetic enzymes of the *tmo* BGC were induced by adding 0.2 mM IPTG. The medium was also supplemented with 250 µM $(NH_4)_2Fe(SO_4)_2$ and 200 µM cysteine after the addition of IPTG. The culture was incubated for another 16 h at 18 °C at 80 rpm before harvesting.

Procedures for purifying TmoA-related peptides from *E. coli* expression

Cells expressing His_6-TmoA or His_6-TmoACys and related biosynthetic enzymes were harvested by centrifugation at 5000 g for 15 min. Cells were resuspended in denaturing purification buffer (50 mM Na_2HPO_4, 6 M guanidium hydrochloride, pH 7.6) and lysed on ice by sonication at 50 % amplitude, 2 s on, 5 s off for a total of 15 min. The lysate was clarified by centrifugation at 49000 g for 15 min at 4 °C. When purifying peptides **1** and **2** containing a free thiol group, 1 mM tris(2-carboxyethyl)phosphine (TCEP) was added to the clarified supernatant. The supernatant was incubated with Ni-NTA resin (Takara) at 4 °C for 10 min. The resin was collected by centrifugation at 2000 g for 3 min. The resin was washed sequentially with 20 mL each of 30 mM, 40 mM and 50 mM imidazole in the denaturing purification buffer. The resin was then washed with 20 mL of non-denaturing buffer (50 mM HEPES, 100 mM NaCl, pH 7.6). Resin-bound peptide was eluted with 500 mM imidazole in non-denaturing buffer. A small aliquot of eluted peptide was desalted using Ziptip C18 and analyzed by MALDI-TOF MS. The remainder of the eluted peptide was concentrated, and the buffer was exchanged to non-denaturing buffer using a 3 kDa molecular weight cutoff filter (MWCO, Millipore). The concentrated peptide solution was stored at -20 °C for future analysis. Peptide concentration was estimated by Bradford assay using BSA as standard.

Procedures for the expression and purification of TmoS

E. coli codon-optimized *tmoS* was cloned into the MCSI of pRSFDuet-1 to produce TmoS with an N-terminal histag fusion with an additional Tobacco Etch Virus (TEV) protease linker (ENLYFQS). *E. coli* BL21(DE3) cells were transformed with pRSFDuet:His$_6$-TEV-TmoS and pGro7, which encodes GroEL and GroES chaperones (4). Cells were selected overnight at 37 °C on LB agar with 50 μg/mL kanamycin and 25 μg/mL chloramphenicol. A single colony from the transformation was inoculated into 5 mL of LB with the same concentration of both antibiotics mentioned above and grown at 37 °C at 220 rpm overnight. The starter culture was inoculated into 500 mL of LB medium with 50 μg/mL kanamycin and 25 μg/mL chloramphenicol and grown at 37 °C with 220 rpm until the OD_{600} reached 0.3. Then, 1 g arabinose (0.2 % *w*/*v*) was added to the culture to induce expression of GroEL and GroES. When the OD_{600} reached 0.6, the culture were chilled at 4 °C for 1 h and expression of TmoS was induced by adding 0.2 mM IPTG. The culture was incubated for another 16 h at 18 °C at 220 rpm. Cells were harvested by centrifugation at 5000 g for 15 min at 4 °C and resuspended in 25 mL of protein purification buffer (50 mM HEPES, 300 mM NaCl and 10% glycerol, pH 7.6). Cells were lysed on ice by sonication at 40% amplitude; 2 s on, 5 s off for a total time of 15 min. The lysate was clarified by centrifugation at 49000 g for 15 min. The supernatant was incubated with Ni-NTA resin for 10 min at 4 °C. The resin was first washed with 10 mL of heat shock protein removal buffer (50 mM tris(hydroxymethyl)aminomethane, 50 mM KCl, 20 mM $MgCl_2$, 10 mM ATP, pH 7.5), and was again washed with 20 mL of 50 mM imidazole in the protein purification buffer. Resin-bound protein was eluted with 500 mM imidazole in the protein purification buffer. The eluted protein was concentrated and, the buffer was exchanged into protein purification buffer using a 10-kDa MWCO. Protein concentration was estimated by A_{280} using NanoDrop. Typical yield of His$_6$-TEV-TmoS was 2 mg/L. The protein solution was aliquoted, flash-frozen and stored at -80 °C for future assay.

***In vitro* assay for the activity of TmoS**

An 100-μL *in vitro* reaction was set up with the following components: 16 μM peptide **2**, 2 μM His$_6$-TEV-TmoS, 1 mM TCEP, and 1 mM SAM in 50 mM HEPES, 100 mM NaCl, pH 7.6. The reaction proceeded at room temperature for 1 h and was analyzed by MALDI-TOF MS. To test whether His$_6$-TmoACys was accepted as a substrate, another *in vitro* reaction was set up using the same concentrations of components except that the peptide His$_6$-TmoACys was used as the substrate.

Preparation of Ti(III) citrate, *E. coli* flavodoxin (flv), and *E. coli* flavodoxin reductase (fpr) for TmoD activity assays

Ti(III) citrate was prepared by mixing 200 μL of 1 M aqueous sodium citrate with 100 μL of 10-15% $TiCl_3$ solution in 12% hydrochloric acid and slowly neutralizing with 50 μL of saturated aqueous Na_2CO_3 solution. The resulting solution was dark purple and estimated to contain 220 mM Ti(III) citrate. The stock solution was imported into a Coy anaerobic chamber to degass and was further diluted with H_2O to 50 mM for *in vitro* assays.

Plasmids for expressing *E. coli* flavodoxin and flavodoxin reductase (pTYB1-EcFlv and pTYB1-EcFpr) were obtained from Squire Booker's laboratory (Pennsylvania State University). Each gene

was cloned as a C-terminal chitin binding domain fusion. Expression and purification of each protein followed previously described protocols (5, 6).

Procedures for trypsin digest and LC-HRMS/MS analysis

To obtain high-resolution mass spectra and pinpoint the position of the enzymatic modifications, LC-ESI-HRMS/MS experiments were carried out. Either Ni-NTA purified peptides or *in vitro* reactions were diluted with equal volume of 50 mM $(NH_4)HCO_3$ to a total of 100-200 µL. Then, 2 µg of sequencing grade trypsin was added and the digest was allowed to proceed at 37 °C for 8 h. The trypsin digest was acidified with 1% formic acid and cleaned up by TopTip C-18 (Glygen) columns. Tryptic fragments were eluted with 60% acetonitrile + 0.1% formic acid. The elution fraction was lyophilized and reconstituted with H_2O for subsequent LC-HRMS analysis. Column used for separation was an Agilent C18 AdvanceBio Peptide Plus 2.1 x 150 mm, 2.7 µm and was maintained at 45 °C for all analysis.

MS parameters were as follows: ion polarity: positive; mass range: 50-1700 m/z; slicer mode: high resolution; gas temperature: 320 °C; drying gas: 8 L/min; nebulizer pressure: 35 psi; sheath gas temperature 350 °C; sheath gas flow 11 L/ min; capillary voltage: 3500 V; nozzle voltage: 1000 V; fragmentor voltage: 125 V; skimmer: 65 V; Oct 1 RF Vpp: 750 V. Acquisition mode: targeted-MS/MS. Acquisition rate: MS^1: 10 Hz, MS^2: 5 Hz. Isolation width: 1.3 m/z. Collison energy: 10 eV.

HPLC parameters were as follows: flow rate 0.4 mL/ min. Mobile Phase A was H_2O + 0.1% formic acid and mobile phase B was acetonitrile + 0.1% formic acid. For analysis of the tryptic fragments of peptides, LC method one was used (see below). For the co-injection assay, LC method two (see below) was used.

LC method one:
1% B at 0-2 min; 1% B to 20% over 5 min; 20% B to 23% B over 5 min; 23% B to 100% B over1 min; 100% B for 2 min. Then the column was re-equilibrate at 1% B between injections for 2 min.

LC method two:
1% B for 2 min; 1% B to 20% over 2 min; 20% B to 23% B over 7.5 min; 23% B to 100% B over 1 min; 100% B for 2.5 min. Then the column was re-equilibrated at 1% B between injections for 2 min.

Data analysis was performed using Agilent MassHunter Qualitative Analysis 10.0. The m/z lists for MS^2 spectra were exported for figure generation using Interactive Peptide Spectral Annotator (7).

Procedures for native ESI-MS analysis

The aerobically purified TmoAD complex was diluted to 1 mg/mL using 50 mM HEPES, 300 mM NaCl and 10% glycerol, pH 7.6. The protein solution was desalted twice into 50 mM ammonium acetate using two 0.5-mL 40 kDa MWCO Zeba spin columns. After desalting, the solution was immediately analyzed by native ESI-MS. MS parameters were as follows: resolution 12500, scan range 500.0 - 12000.0 m/z, microscans 10, maximum injection time 50, advance gain control target 5×10^4, capillary temperature 200 °C, trapping gas pressure 3, in-source trapping on, desolvation

voltage -150 V, S-lens RF level 200. Data analysis was performed using BioPharma Finder software and the ReSpect algorithm was chosen for deconvolution.

Procedures for generating peptide standards containing different butyl group isomers

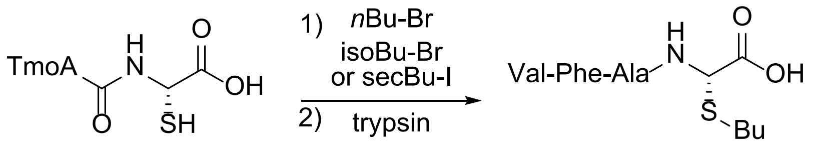

To 10 μL of 160 μM peptide **2** in 50 mM HEPES, 100 mM NaCl, pH 7.6 was added 0.4 μL of 100 mM TCEP. After brief mixing, 20 μL of 5% solution (*v*/*v*) of the corresponding butyl bromide/iodide in acetonitrile was added. The reaction was allowed to proceed for 8 h at 37 °C with shaking at 220 rpm. The reaction was centrifuged at 13000 rpm for 5 min and the supernatant was lyophilized to dryness. The lyophilized residue was resuspended in 150 μL of 50 mM $(NH_4)HCO_3$ and was digested by 2 μg of sequencing grade trypsin at 37 °C for 4 h. The trypsin digest was acidified with 1% formic acid and cleaned up using a TopTip C18 column. Tryptic fragments were eluted using 60% acetonitrile + 0.1% formic acid. The elution was lyophilized to dryness and reconstituted in H_2O. The solution thus contained the standards of the VFAX peptides (X denotes the last amino acid with different isomeric butyl groups).

Procedures for the *in vitro* reaction of TmoD using d_3-SAM
A 50-μL reaction was set up anaerobically in 50 mM HEPES, 100 mM NaCl, pH 7.6, containing 23 μM peptide **3**, 14 μM reconstituted $TmoA_{37mer}D$ complex, and 1 mM SAM or d_3-SAM. The reaction was initiated by adding 1 mM Ti(III)citrate (final concentration). The reaction proceeded in the dark at 16 °C for 12 h. The reaction conversion was first checked by MALDI-TOF MS and showed largely trimethylated product with minor di-methylated intermediate. Trypsin digest was performed in the dark at 37 °C for 5 h. The digests were cleaned up and analyzed by LC-ESI-HRMS/MS as described above.

Procedures for the *in vitro* reaction of TmoD using d_5-peptide 5
The peptide d_5-**5** was prepared similarly as described above by reacting peptide **2** with d_5-EtI. A 50-μL reaction was set up anaerobically in 50 mM HEPES, 100 mM NaCl, pH 7.6, containing 100 μM peptide **5**, 17 μM reconstituted $TmoA_{37mer}D$ complex, and 2 mM SAM. The reaction was initiated using 2 mM Ti(III) citrate (final concentration). The reaction proceeded in the dark at 16 °C for 18 h. Trypsin digest was performed at 37 °C for 5 h. The digests were cleaned up and analyzed by LC-ESI-HRMS/MS as described above.

Procedures for testing the protease activity of TmoG
E. coli codon-optimized *tmoG* was cloned after the NdeI site of pET15-b to generate a fusion protein with an N-terminal histag with a thrombin cleavage site. The construct was used to transform *E. coli* BL21(DE3) cells. Cells were selected overnight at 37 °C on LB agar with 100 μg/mL carbenicillin. A single colony from the transformation was inoculated into 5 mL of LB with 100 μg/mL carbenicillin and grown at 37 °C at 220 rpm overnight. The starter culture was used to inoculate 500 mL of LB with 100 mg/L carbenicillin and grown to OD_{600} 0.6-1. The culture was chilled at 4 °C for 1 h and protein expression was induced using 0.2 mM IPTG. The culture was

incubated for another 16 h at 18 °C at 220 rpm with a final OD_{600} of 2.6. Cells were harvested by centrifugation at 5000 g for 15 min and resuspended in protein purification buffer (50 mM HEPES, 300 mM NaCl and 10 % glycerol, pH 7.6) with a ratio of 3 mL of buffer per 1 g of cells. The cell suspension was flash frozen and stored at -80 °C for future activity testing.

The cell suspension was thawed and lysed by sonication on ice: 20 % amplitude, 1 s on, 9 s off for a total of 8 min. Since TmoG is membrane-bound, whole cell lysate was used to test activity on different TmoA-related peptides. The lysate of cells overexpressing TmoG (500 µL) was first diluted with 250 µL of protein purification buffer supplied with 100 µM $ZnCl_2$. To initiate the cleavage reaction, 40 µL of peptide stock (10 µM final peptide concentration) was added. The cleavage reaction was incubated at room temperature for 24 h.

TmoA-related peptides from each cleavage reaction were purified before analysis by MALDI-TOF MS. Each cleavage reaction was centrifuged at 14800 rpm for 5 min and the supernatant was incubated with 150 µL of Ni-NTA resin at room temperature. The resin was collected by centrifugation, washed sequentially with 20 mM imidazole and 40 mM imidazole in 50 mM HEPES, 100 mM NaCl, pH 7.6. Resin-bound peptide was eluted using 2× 200 µL of 500 mM imidazole in 50 mM HEPES, 100 mM NaCl, pH 7.6. Each elution fraction was desalted using a C18 Ziptip and analyzed by MALDI-TOF MS.

***E. coli* codon-optimized DNA sequences encoding TmoACys, TmoB, TmoH, TmoI, TmoS, TmoD and TmoG**

tmoACys

```
ATGACCGAACGCGATGCGACTGCTACGGATGAACTGGAGCCGGTTGCAACGGAAGCGGAAGGGA
CCACCGATTCGACGGCTGCCACTGATACCGCAGCGACCGACACGGCAACGGACGGCGCAAAAAC
CGTCGAAGCTGACGCCCTCGAAGCAGATGGCTTGGAAGATTTTGACTTGGAAGATGTTGAGATT
ATCGAAAGCAAGGTGTTTGCGtgcTAA
```

The lower-case tgc indicates the codon for the C-terminal cysteine in the peptide sequence.

tmoB

```
ATGACCGTGGCGACGCGCCCTGCACCGTCAGCAACCCGTTTGGCGACCGCGGATCAAGCCGCCA
CGACCGACCAGGTCAGTACCACCGGTCAAGCCAGCGCAGGAGATCTGCCTGCGATTGACTGGTT
CCCACTGTTTTGGATGCGTGGCGCGGGCTTTGGTTTCGCCCGCCTGCGCTGCGCGTGTCTGGAT
GCCGACATGTTAGCGGTGATTGATGACCCGGCAGTTACACCAGATTCGCGTGCGGCGGCGTTTG
CGCCTGCGGCAGCCGACGCCCGCCGTCGCCTGGTCGCGGCCCTGGGCGACGCCATGGCCGAGGA
AGCGTTATTTCTGTCCAATCGCGATGCACCAGCCCGCATCGCGGAGTTAGCCGCCGAAAATCTT
GATCAGCCGCGTAAACGCGCCCGCCAAAAACTGCGTCTCGCATGGTCCTATCTGCAACGCTTTG
CGGCGAAAAACGATACTGCCTCTTTCTTTGGCCCAGTGGCCTGGGGCCGGATCGATCCTCGTTC
CGAGACCGCCCTGCAGGTGACCGGCGACCCAGATCGCGTGCCCGATCGCGTAATTGGGGATCGC
GTGATTGGTGACCGCATCACGGTCATCGAGCACTGGGTTGCCGAAGCATTAGCCGAAGCCGTGG
CAGCCGACCCTGCATTGGCAGGGCAGCTGCCCTTGATCCTCAATCCAGGCTGTACCCTGGACGA
TGCAGGTCGTTTACGCTTGCCAGTTGATCGCTCGACCCCCCTCCCGGCTCGCGTTGCAGCGGTC
GTGCGTGCGGCGATGCGCTTGGGGCCGGCGCGCCCTGCGGGTCTTGCGGCAGCTGCCGCGACCG
GTGATGCCGCTGCAGCCATCGATATGTTGGTGGGCCGTCGTGTGTTGTTGCCGGCCCTGACACC
GGCGCCGGGATCCCGCCGCCCGTTAGATCATTTAGCGGGTCTTTTGGGGCGCATCGGAACGCCA
```

GCGGCGGACTTATGGCTGGATCGCATTGCAGCGATTGAAGCGGGCGCCCGGGATTTTGCCGCCG
CGGCCGGCCGCCCGCGTGACCGCCTGGCGATTCTGGATCGTCTGCACACCTTGCTGGCGGCGGC
GGGCGCCGATCGTGAACGCGATCACGGACGTATGTACGTCGGCCGTCAGCCGGTTTATGAAGAT
TGTGCGCGCGCTGGTAACGTCGTGTTGGGCGGCCCATTTCTGAAAGCTGCCGAGCCGGATCTGG
CCCCAGTACTGAACCTGTATCGGCGTGTCGCTACGGCGGTGGCCATGTTGATTGCGGATGGCCA
GGCACAAGCCTTTGATCGCCTGGCCCACGATGGCGCGGCGGATGCAGGCGGCACCGTTGATCTG
GTTCGTCTCCTTGCGGCGACCCCGTCAGCGGATCTGGCGACCGCCGCGGTTGCTGTCATTGAAC
CGGTTCTCCGTCAAGCCTGGGATCGCCTGGCGACGGACGCTTCCGTGGACGAAATTGCGCTGAC
CCCTGCCGATATCGCCCATGTCGGCGACGCCGTGGAGACGGCGGTTCGGCGCCTGTTGCCGCGT
GCTCCCCAGCCTGTAACCCTGGGTTTAGATATCGCATCGCCTGATCTCATGATCGCTGCTGCGT
CGCCGGCCGCTATCGCCCGTGGGGACTGGCGTTTAGTGATCGGGGAGGTGCATCCGGCTGTGGC
GACGGCGGTGCAACCAGTCGCCATGCCATTCTGCCCGGACCCCGATCAGGCCGCGGACATGGCG
GCCGGCTGGGCCTTAGACGGGGCTGCCGGCCCGGCACGTCTGGGCTTAGCGGACGATGGCCGCC
ATTACCAGCGCTCCCATATTGATTGGCCGGAGCACCCAGCGTTCATTGATATTGCTCTGCCTGG
GGACGCGGCAACGGGGCCACGTCGCCGCATTCGGAGCGCGGAAGTGCGTGTGCGTCGCGCAGCA
GACGGCACGCTTCGCATTCGTAGCCGTTGTGGCCAGGACGACGACCTGGGGACCCTGACCCGTA
CAGCGCTGCATCGTGCGATGTTCACCCTGGCCTCTGCGGTTACAGGCCGTCATCTTGCGCCGCG
TTTAGTGTTAGGCCGTCTCGTTGTAAAACGTCGCTCTTGGCAAATTAAAGCAGACGATCCACGT
CCGGGCGGTAAGCCCGCCGAAGATGCAGAAGCATTTCGTGCAATGCGTCGTTGGGCCCGTGACT
TAGGGCTTCCACCGGCGATCTTCGCGAAAGCCCCGGGCGAGCCGAAACCGGTATGTATCCTGCT
GGATGCCCCACAGGGAGCCGAGATGCTGGCCCGCCTGTTTGACCGGGATGAACCGATTGATCTG
GCTGAAATGAGCCCGGGTCCGGATGAATTATGGCTGGATGCGGGGCCGGAGGGCCGTGTGACAG
CCGAATTCCGTCTGAGCACCCGCCTGCGTCCGGCAGCCCCCACTAAAGATCCCGCGTAA

tmoH
ATGGATAAACCGGCACCGCTGGGAGTGGGTTTGCAGTATAACCCGGAAATCTTGGATTGGTTCC
CCTTTGAAACCCAACCGGTGGATCTGTTCGAGATCCTGCTCGATGCGATTATGGGACCTTTAGA
TAGCCCGTGGATTTTTCGCCCGGGTCAGGAACGCCGCATGCAGGCATTGGGGGCAGCAAAACCA
CTGCTGGCGCACTCGAACTATGGCTGCGAGTTTGGCTTCGGACGCTTAGAGGATACTCCCGCAG
TCCGCCGTCACGTTGCACTGGCGAAAGCCATCAATAGCCCCTGGGTCGGGGATCATTGCTTTTA
TGGCGATGGTTCATGGCTGGATATTTGGTCTAGTCCCGTCCAGTTTTCGTCTCCAGAACTCGTA
CGTGTTGCGGACCGTGCCCGCCGTCTGCAGGATATCTATGGTATGCCGTTAGCCCATGAAAATG
CGGCTTATTACATGCCAACCCCTGGGGCAGCCATGCGCGAAGCAGAATTTATGGCGCGTTTAGT
CGACCGTGCTGGCACATGGTTGCATCTGGACTTACATAATGTGTATACCAACTCAGTTAATCTT
GCCGGCTATGACGCGTCAGATTATCTGGCGACGATTCCACTGGACCGCGTGGTGTGTATCCACT
TGGCGGGTGGCAGCTGGTATGATGGTTTGTACCATGATTGGCATGACAGCACTGTGCCGGAGCC
GGTATGGGCGATGCTGGACCAGGTCCTGTCACGTGCGTTACCGAGTGCAGTCATTCTGGAATTT
CAGGGCCGCGCACACCACCCACAAACCCGCGAGTTGGGCGGCGCGGAAGATGAAGCGATGATCG
CGGCAGACCTGGCGCGGGCGAAAGCGGCCTGGGATGCGGCAGCCCGTCGCCACGGAGTAGTACC
ACCGGTGCGGGAAATCGCATAA

tmoI
ATGACTGCGATGACTCCTCCTGCAGCGCCAATTCCAGGCGCCGGCCTGCCTGCCGCAACTGTTT
ATGCGACGTGGCGTCGTATTTTACGTGAAGCGCCTCTGGCTGAGGCAATGTTTGATCCGGCCAT
CGATGACCAGACCCTGGGGCGTCGCTTCGGCCTGGATGACGATGGGGTGGCCACGGTACGTGCC

TATGCTGCGACCCCAAAACCTACCCGCATGTTTATCTTGAACTATCGGTTTCGGATGACAGGGT
CCGTACAAAACGCTTTAGAGACCGCGGCACCGCTTACTATGCGCGCTCTGAAGGCAAAAGGTCT
GGATTTACGCGAACTCGCCGAAGGCTTTTTGGAAGCCGATCATTGGCAAGATGATGGTCCGTTC
GTGGTGGGCTACTGCGCGCGTATTCTGGACCACCTTGCTGGGGATCCGGCCACAGAAGCGCCGG
CAGGACTGCGTGATCTGATTGCACTGGACCGCGCAACGGCCGGACTCTTAATGCGTCTGGCCGA
TGCGCCACCGCAGCCGGCCGTCCCGGCTGGTCACGTAGGTCTGACGGGCCGGGCAGTCGCTGTA
ACCACGGCGCATGATCTGGCGCCTTGGTTACGTAATAGCGCGGCGTTGGGGCGTGAAGATCTTC
CGGCGCGGCCTGCCGCGTATCTCGTGGCAATGCCGGACCTTGCAGCGGCCCCGAAATTGTCGGC
GCTGCCACCGCGCGGCGCGTTGATGTTGGCAGCGCTGGAAGAAGGCCCACTGTCTCCTGCCGCG
CTGACCCGTCGTTTGGGTGGTGCTGGTCCGCAAGACGCGGCTTTACTTGGTAAATTGGCCGAAC
GCGGTGCGGTGGTTGGCGCCTAA

tmoS

ATGACTGCAGCACCTACAACTGACGGAGCGGCCGCAACGTATGGTTCACGTACTCACTGGGATG
GGGTTCATGCCTTGCTTGAGGACCGTGCCTTACTGGAAGATCGCAGTGGCGCCCGTCCGCAGGC
GGCCGGAGCACTCCTCGACCATATGGCCGCAGTCGCAGACCGTTTAGCTGGAGCAGGCGGGGTT
GTTATGGACTTGGGCGCGGGCCAAGGTGCATTAGTACGCGCCCTCGCCCAACCGGGTCGTTTGG
TAATTGCGAGCGACATCGCCGCAGGACCCCTGGCGGCAATTCGTGTGCCTGGTATTGCTGGCTT
GGTTGCCGACGCGGCTCGCCCGGCCCTGAAGCCTGGCGTGATCGACCTGATCACGTGTTTACGT
GGCCTCTGGACGCTCCCTGACCCTGCCGGTGTTTTAACTGCCATGGCCGGGTTGTTGGCTCCTG
GCGGACATATCCTTGTTCAATTGTGGGACCGCCCAGCCCGCTGTCGTCTGATCGGCACGGGAGC
CGCTCTCTTGGGCAAGGTTCTGCCCGGTTTAACGCGCCCGGCCGGTGAAGCTGGTCCGTTCGAT
ATCGACCCAGATGGTCTGGTGGCGCTTGCGGCCCCTGCGGGCCTGGAATTAGTTGCAATTGAGG
ATGGGGCAGCAACTGCTGGGATTTGCGATGCCGCTGGTTACTGGCGTGAGTTCGATGCGTTAGC
CGAAACTGCGGCGGCGGCACGCCTTCGTGCACCAGCCGCGCTGCAGGCACGCTTGGACGCGGCT
CTGCCAGGCGTATTAGATCGCGTACTCCCTCGCGGTGGTGATGCTGCAGTGTGGCAAGCGCCTA
TCGCGTGGCACTTGTGCACACTTGCCCCTCGTACTGTGCAAGACCGCATTACTCCCCCTGGCTA
A

tmoD

ATGACAGGTCACTTTCGTATGAGTCAAGATCCGGATCTGCTCAGCCCTGACCATCAGGGCATGG
ATATGCCGGACACCATGATTGCGCGGACTGCGACGGCAGCCTCTGCTGGCTTTGGCGCGAAACC
GACCCTGCGTGTGGGCCTCGTAGCGTATAATTGGGAAGCGGATACCTCCTTGGCTTTGTGGAAT
TTGGTGACGTACGCTCGCCGCACCCCTGCCATCGCGGATCGGGTGACCTTTATTGAACACTGTG
CCGCGACCCCCAAGAGTCTTATGGACGAAGAAGCGCAGCTGTTTTTCCTGCTGAAATGGCAAGA
GACGCACAACTTCGATGTGGTTGGATTTTCGTGCTATATTTGGAATATCAAATTTGTACTGCGC
GCTGCAAAAGCCATTAAAGAACTGTGGCCGCAGACCGTCGTCATCTTCGGTGGCCAACAAATCC
GTGGTAGCTACGTTCCGTTTGTCTTCGACCGTGAGCGCTGCGCGGATATTTGCGTGTGCAATGA
AGCTGAAGTTACCTTCCGCGATCTTTTAAACAGCTTTCTGGATGGCAGTCCAAAACTGAGCACT
GTTGGTGGGATTGCCTATATGGCTCCAGATGGTAACACGGACGACTTTCTTATCTTTGATGGCT
CAGGCCGTGTCCCAGCAGGAACCGCCTATCATGAGACGGCGGAGGCGGCCTTGATCGAAGACAT
TAACGATATCCCATCTCCTTATTTGGGGGGTATCGAACTGCCCCACGGCGGTGCGTTTTTATAC
GAAGCGAGCCGTGGCTGCCCGTACCGTTGCAGCTTCTGTATCTGGGGTGAGAGTAAAGGTGTTC
GTGAATACGAAATGGAACGTGTTGAAGCTGAATTACACAATATTCTGAGCCATCATCCGTCACA
TATTATGTTTTGCGATGGTACTTTTAATATGCGTAAGGAACGTGCAGCACGCATCCTGGAAATT

CTGGTCGAACATCTGCGCGATGGGCGCGTCCAGCCGTTCAGCCTGCTGCTGGAATTAAAGCTGG
AGATCATTGATGATAATCTGGCACGGGTGATGGATGAACTGGTACGCTTAAATCCACTGGTCAC
GTTCGAATTTGGTTTGCAGTCAGCGAGTCAGGAAGCCGCGAAACTCATGCGTCGCCCGTTTACT
GAAGATCGTTTCCGCCGTGCTTGGAATCGCTTAAGTGATAAACTCAAAAGCAGCGCGATCATTG
ATTGTATCTATGGGCTGCCGGGTGACGGAATTCCTCAATTTATGCAAACCGTCGACTTCTGCTA
CAGTCTGTCACCCCATCGCATTCAAGCTTTTCGCCTGTCAATCCTGCCTGGGAGCGAGTTTGAA
CGGCAGGCTCAAGAGCATGAAATCCGCTACATGCGTGAGCCTAACCATATGGTGTACGAAACTA
AATGGCTGAATCTGGATCAGATGGCCTGGGTAGAAACATTCGGCTTTGCCGTTGCGGACTTGTA
CCACTTCCATGGGACCACTATTAAGTGTCTGCTGGCGATGAAAGATCAGTTAGGCGTCGACGGT
TTCTCTGACCTTATTCGCCGTTTTGTGGATTGGGCTGGGCGCGACCGTATTCTGGGGAGCATTT
ATGCCGGGAATCGCCCGGAAGGTCGGTGGCGCGCCATCGACCTGTCTAAACCGTTTGAACAATT
TATTCAGACGCGTTTGCTCCCGGACGCCGGGATCACCGATCCCTCGCTCCGCGACCGCTTGCAT
GACCTCTTCCGTTACGAAGTGACCATGGGGCGCGTTGCGGTCGGTGGCTTAGCAGAGACCCCAC
TGCCAGCGTTGTCCGCGGGGGGTGGCGCGGGCGAGCTGGCGATTCGCGTTGAACTTATGCACAG
TCGCTTTGATATTCCAGGTTTCATTATGGGCGCGCATGGACGTGGGGACATCGATATTATGGAT
ATGGATGAGACGGATAGCTACGTGGCCTTCACTCAGAAAGCTGGACACCAAGGCGTGTTAGTGC
CGATTTCCTATCGCATTTCGGGTCGTATGGCAGGCTTGATTGAAGCGGCCCGGCGTGACACGGC
GGCGGGGGCCGGACTGGCACCAGTGCTGAGTAAACTTGTTAAAATTGGAATTGTAGCGGAGGCG
ACCGGTTCCTAA

tmoG

ATGACGACCCTGACGGCCCCCGAAATTGACACGACTGCAGCTGAAAGCGCTGGTGCCGCTATTG
CCCGCGGTTGCTTCCCTGCACGTTTCGAGCACGCCGAAATCACTGATCCTGCCCGCGGTCGCCG
GATCGAAATTACAGACCCAGCCGGAGATGGTCACTGGGTTCTGGGCCCGGCAGAATTTGAAGTA
GCCCGTGTCTTTGACGGCCAGTCATCGTTTGGCACCTTGTCGGCGCGCTTGGCGGCACGTGGGA
TTCGCGCCGACGCCGCAAAACTGATGCGGTTTGAAACTAAGCTTCTGGGCCTGGGTCTTCTCGA
AATTGATGGTCGGGGTAATCGTGCCCGCGACCCCTTCACAGGTTTCGATTTCGCTCTGCTCCAC
CATTTGGTTATCCAGCGGCTGGGGGTTGTACATCCGGAACCACTGCTGGATCGCTTACTGGCGT
GGCCCGGCGCCGTTCGTATGGGGTTGTTAGGCGTGGTGACCTTGGCGGCTCTCGCCACCCCAGT
ATTGCTGATTGTGATGGGTGGTCGTTTCCTGGATGAGGCGTCGGCTACCTTAACCGGTTGGATG
CTGCCACTGTTGTACTTGGTCACCCTGGCCTCTGGTTTTTTGCATGAAGGGGGTCATGCGCTGG
CGTGCCGCGCTCTGGGCGTGCGTGTACGTGAAACCGGTTTTGCGATCTATTTTCTGATGCCATT
TGCCTGGACCCGCCCGGACCGCCGTGATTGGGAAGCGCTGGCGATGGGTCCGCGTATGGTCGCG
ATTCTGGCTGGGCCGTTTGCGAGTCAGGCGCTCGCTGGGCTTGGCCTGGCAGCTATGATGGCTG
CCCCTATGGGTTCGCCTTTACATAGCGGGGGGGTTATCTTGGCGGCCGCCGGCCTGTTTGGTGC
CACTGTAACGCTGTTGCCAGTCTTAAACGGGGACGGGTACCTGATGCTGGTCGAAGTGCTGCGT
CTTCCCAATCTTCGTCGTCGGGCATTCGACCATCTCCGGCGCGCCGTGGGCTTACCGGCGCCGG
CTTTAGCCGGACCGCTGCCCGCTGGTCGTGGTCCGTTGCTGTTGGCCGTCGCGCTGGGCACGAT
TCTTGGATGGGCGGGTATCTGGGTGGGTATGGCCTGGTGGCTGGGTGGCATGATGTTAGAATTA
ATCCGCTAA

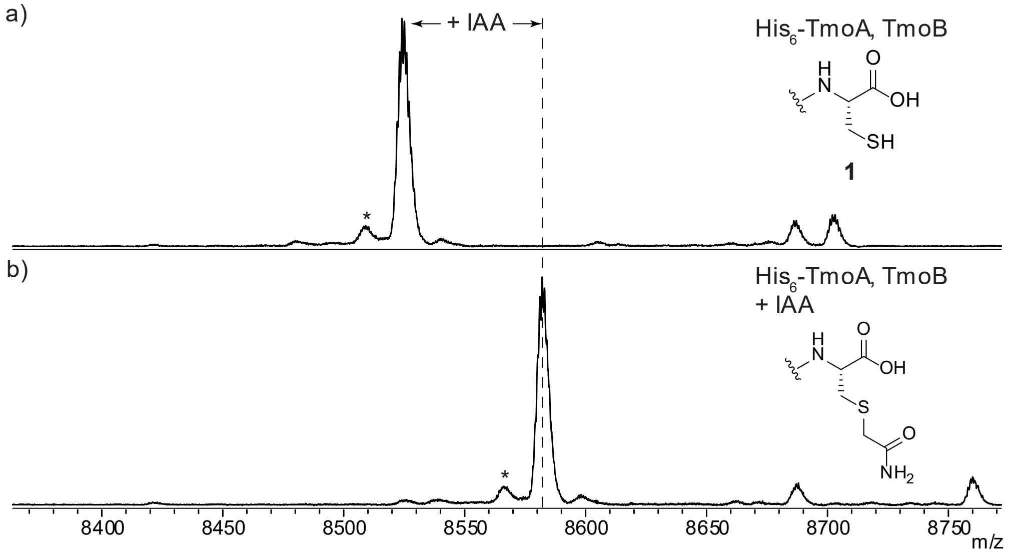

Figure S1. TmoB appends cysteine to His₆-TmoA in *E. coli*. a) MALDI-TOF mass spectrum of the coexpression product of His_6-TmoA and TmoB. Average m/z $[M+H]^+$ for His_6-TmoACys calculated 8528, observed 8525. b) MALDI-TOF mass spectrum of the co-expression product in panel a reacted with iodoacetamide (IAA), indicating the presence of a thiol group. Expected average m/z $[M+H]^+$: 8585 observed: 8582. Peaks labeled with * are deamination artifacts in MALDI-TOF MS.

a)

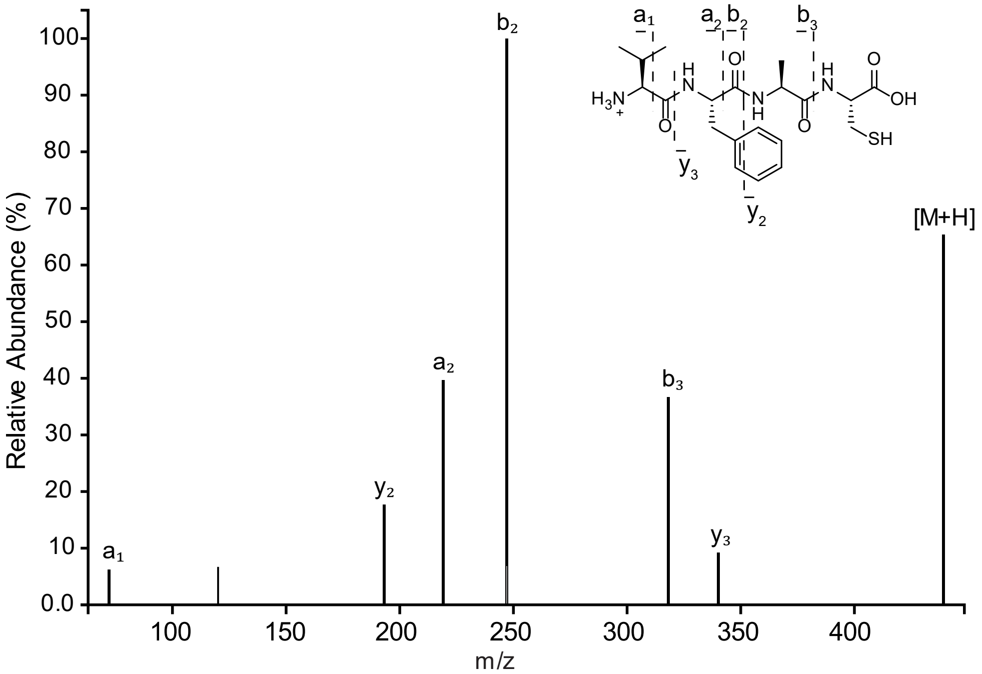

b)

Ion	Exp. Mass	Theo. Mass	Error (ppm)
a1	72.0812	72.0808	5.8844
y2	193.0638	193.0641	-1.7878
a2	219.1488	219.1492	-1.7607
b2	247.1440	247.1441	-0.4065
b3	318.1811	318.1812	-0.3598
y3	340.1318	340.1326	-2.2202
M+H	439.2012	439.2010	0.5256

Figure S2. TmoB appends cysteine to the C-terminus of His_6-TmoA. a) ESI-HRMS/MS of VFAC peptide generated from the trypsin digest of peptide **1**. b) Theoretical and observed ions in the MS^2 spectrum.

a)

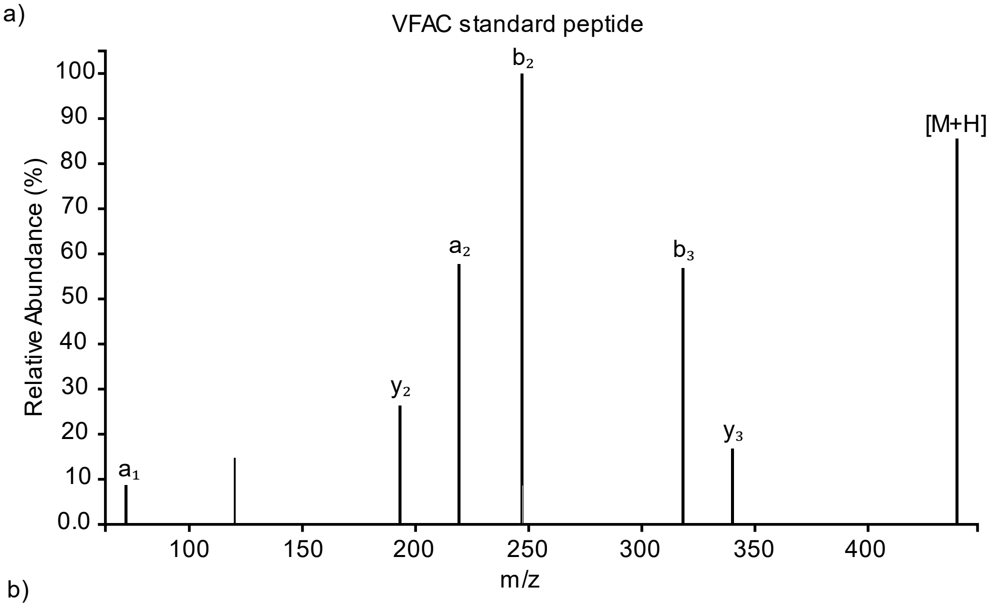

b)

Ion	Exp. Mass	Theo. Mass	Error (ppm)
a1	72.0811	72.0808	4.4971
y2	193.0642	193.0641	0.2841
a2	219.1494	219.1492	0.9772
b2	247.1452	247.1441	4.4490
b3	318.1817	318.1812	1.5260
y3	340.1326	340.1326	0.1319
M+H	439.2020	439.2010	2.3471

Figure S3. MS/MS spectrum of VFAC standard peptide is identical to the MS/MS spectrum of the VFAC peptide generated from the trypsin digest of peptide **1** (Figure S2). a) ESI-HRMS/MS of VFAC standard peptide generated by trypsin digest of His_6-TmoACys. b) Theoretical and observed ions in the MS^2 spectrum.

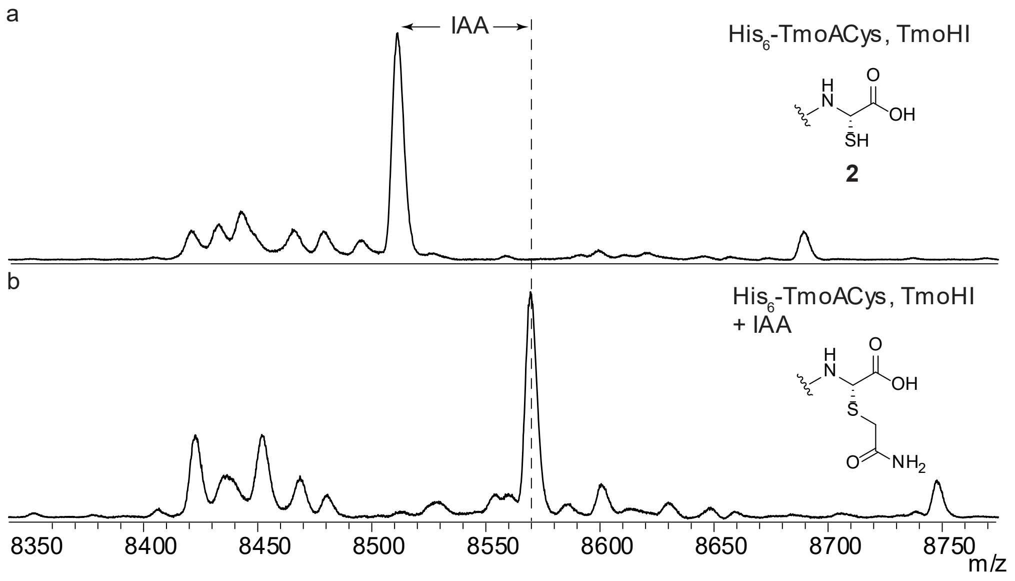

Figure S4. MALDI-TOF mass spectra of peptide **2** and peptide **2** labeled with IAA. a) Peptide **2** generated by coexpressing His6-TmoACys and TmoHI in *E. coli*. Average m/z $[M+H]^+$ calculated: 8514, observed 8511. b) Peptide **2** labeled with IAA. Average m/z $[M+H]^+$ calculated 8571, observed 8570.

a)

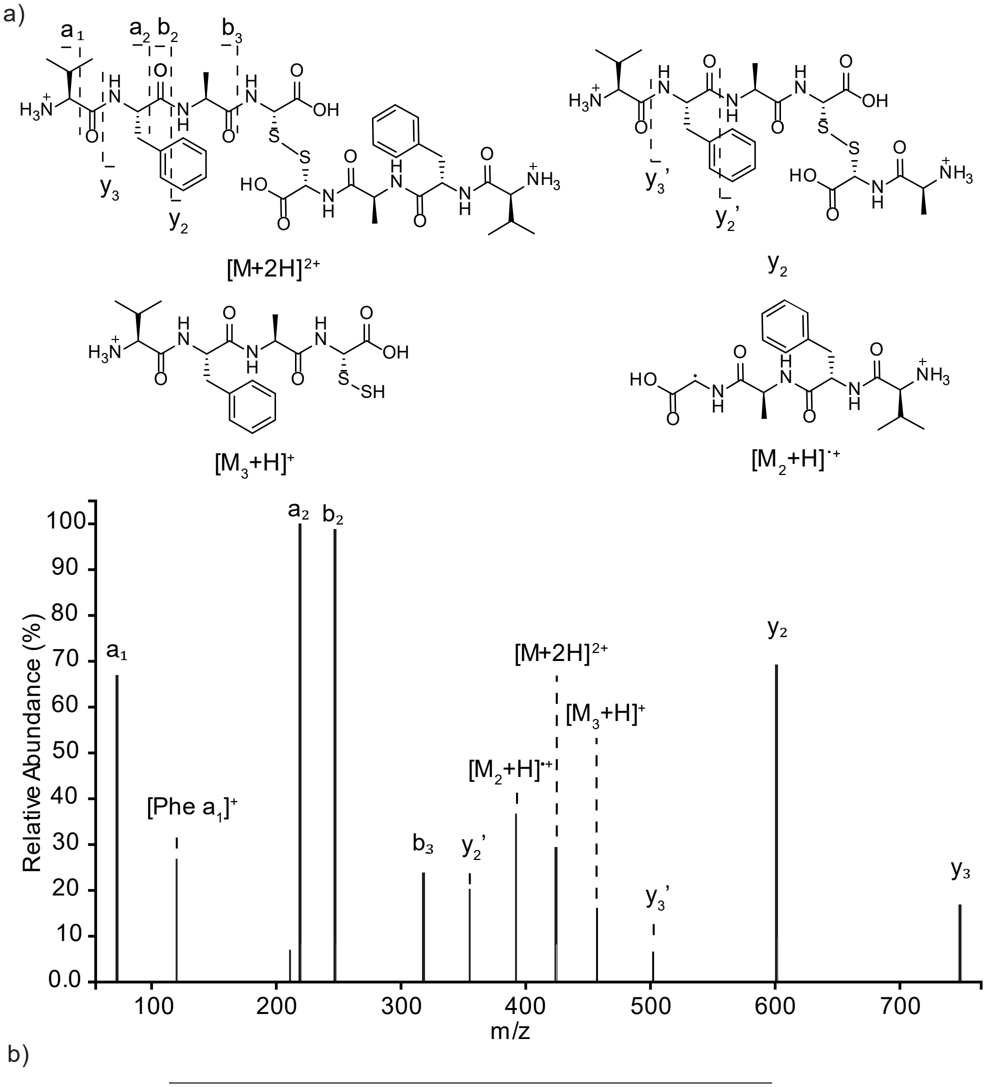

b)

Ion	Exp. Mass	Theo. Mass	Error (ppm)
a1	72.0810	72.0808	3.1097
a2	219.1493	219.1492	0.5209
b2	247.1442	247.1441	0.4027
b3	318.1812	318.1812	-0.0455
M+2H	424.1778	424.1775	0.7313
y2	601.2107	601.2109	-0.2980
y3	748.2789	748.2793	-0.5201
Phe a1	120.0809	120.0808	0.9993
y'2	355.0741	355.0741	0.1408
y'3	502.1428	502.1425	0.6572
$[M_2+H]^{\cdot}$	392.2053	392.2054	-0.3060
M_3+H	457.1571	457.1574	-0.6344

Figure S5. TmoHI excised the β carbon of the C-terminal cysteine in His_6-TmoACys. a) ESI-HRMS/MS spectrum of the disulfide form of the C-terminal 4-mer peptide generated by the trypsin digest of peptide **2** and proposed assignments of the observed ions. b) Theoretical and observed ions in the MS^2 spectrum.

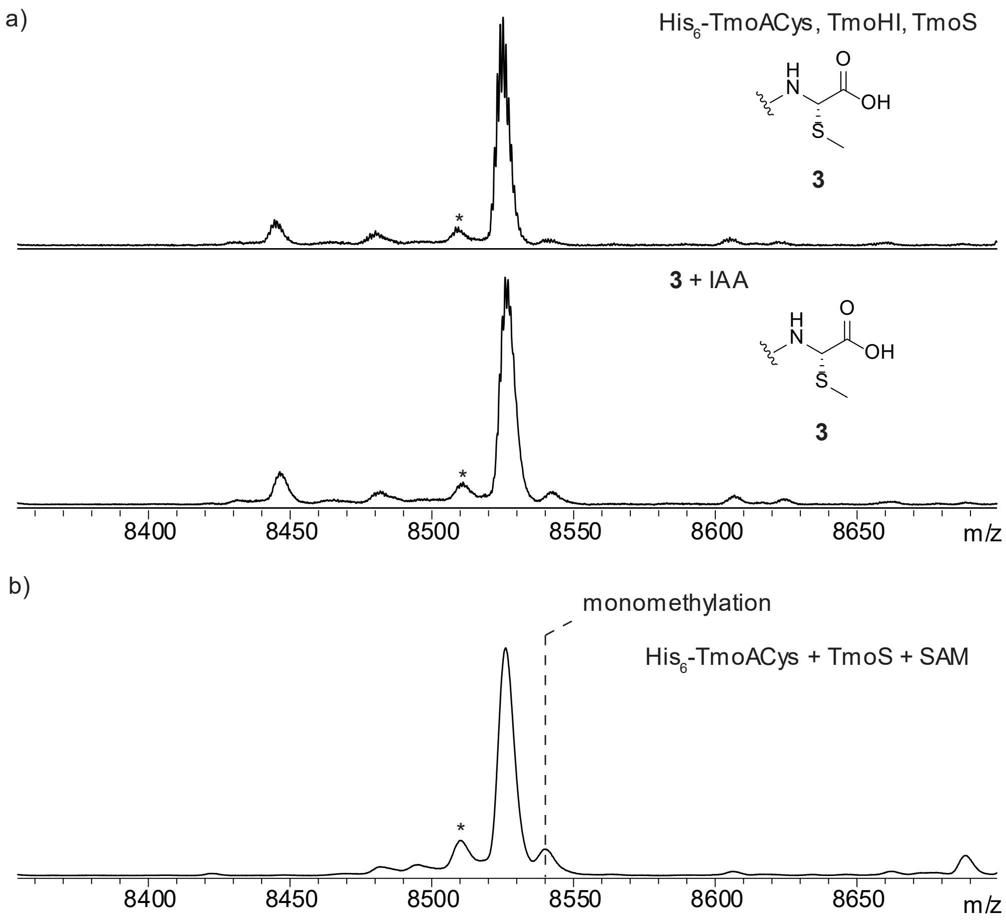

Figure S6. Coexpression of His$_6$-TmoACys, TmoHI and TmoS in *E. coli* generated peptide **3**. a) MALDI-TOF mass spectra showing peptide **3** produced in *E. coli* did not react with IAA. Top panel: peptide **3** produced in *E. coli*. Average m/z $[M+H]^+$ calculated: 8528, observed 8525. Bottom panel: treatment of **3** produced in *E. coli* with IAA. Average m/z $[M+H]^+$ calculated: 8528, observed 8526. b) Treatment of His$_6$-TmoACys with His$_6$-TmoS and SAM *in vitro*. No robust methylation activity was observed.

a)

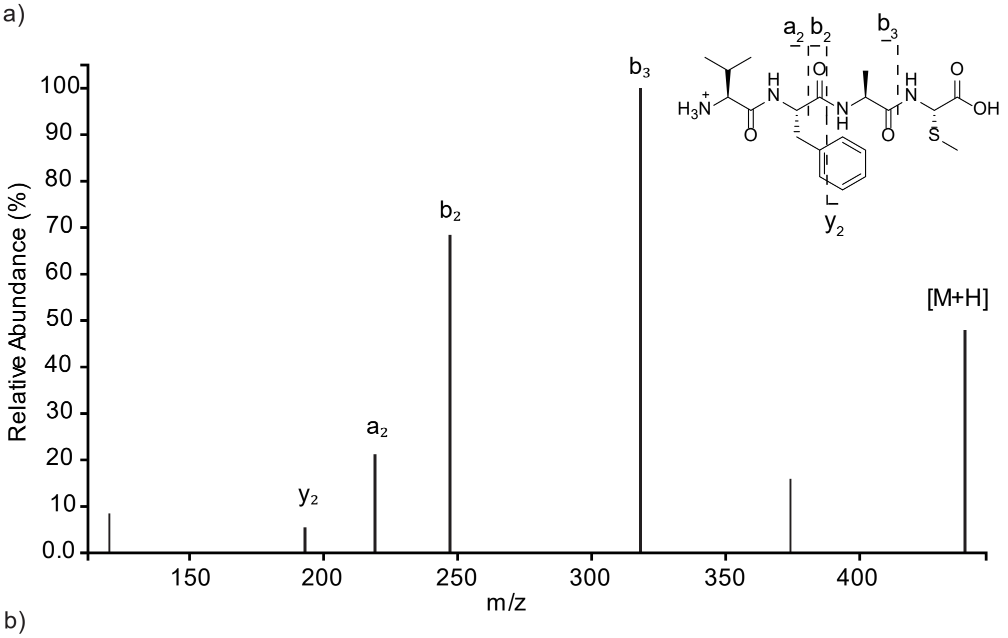

b)

Ion	Exp. Mass	Theo. Mass	Error (ppm)
y2	193.0642	193.0641	0.2841
a2	219.1492	219.1492	0.0646
b2	247.1438	247.1441	-1.2158
b3	318.1812	318.1812	-0.0455
M+H	439.2009	439.2010	-0.1574

Figure S7. TmoS methylated the thiol of the C-terminal residue of peptide **2**. a) ESI-HRMS/MS spectrum of the C-terminal 4-mer peptide after trypsin digest of peptide **3**. b) Theoretical and observed ions in the MS^2 spectrum.

a

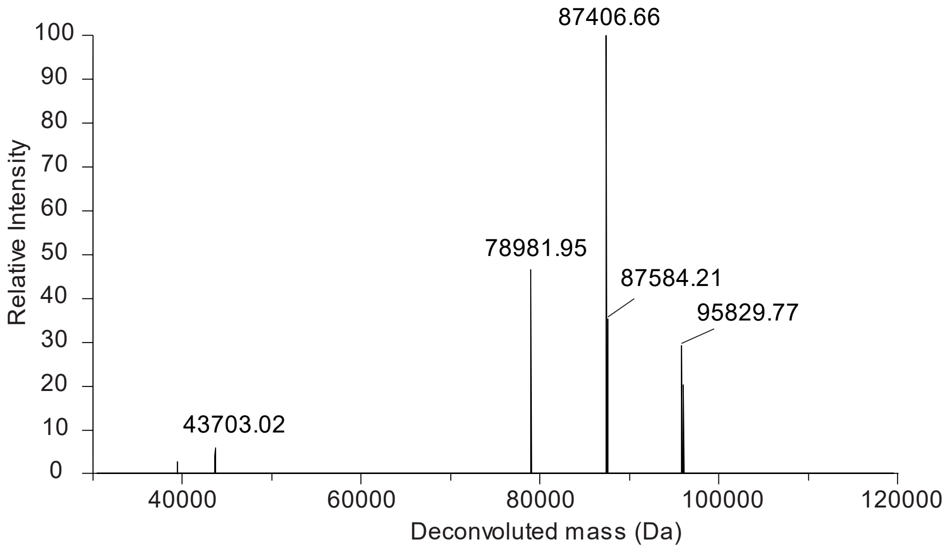

b

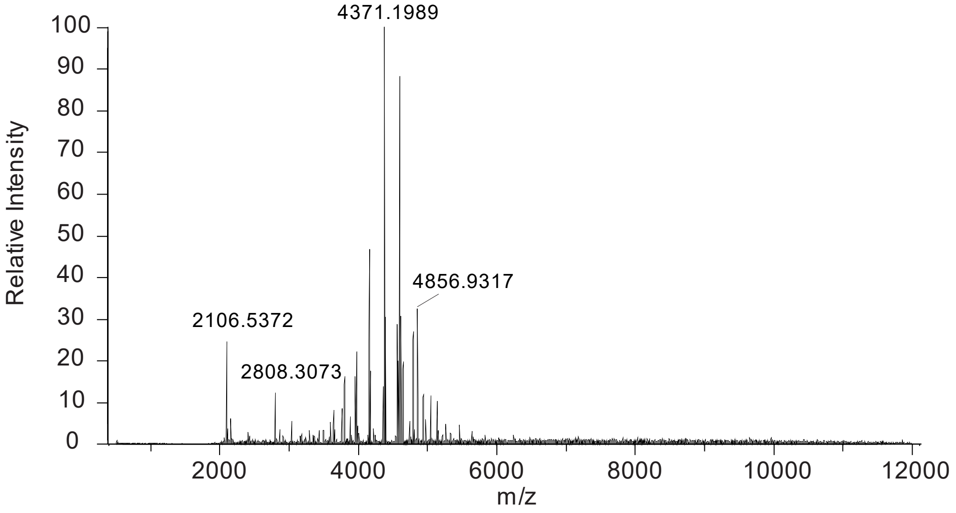

Figure S8. Native ESI-MS analysis of a TmoAD complex. a) Deconvoluted spectrum showing complex formation between His_6-TmoA and TmoD. TmoD calculated average mass: 78983.3 Da. TmoD observed mass: 78982.0 Da. The difference between the complex and apo-TmoD is 87406.66 - 78981.95 = 8424.71 Da and matches the calculated average mass of His_6-TmoA (8423.78 Da). This observation demonstrates the binding of His_6-TmoA to TmoD under the experimental condition. b) Mass spectrum of the TmoAD complex before deconvolution.

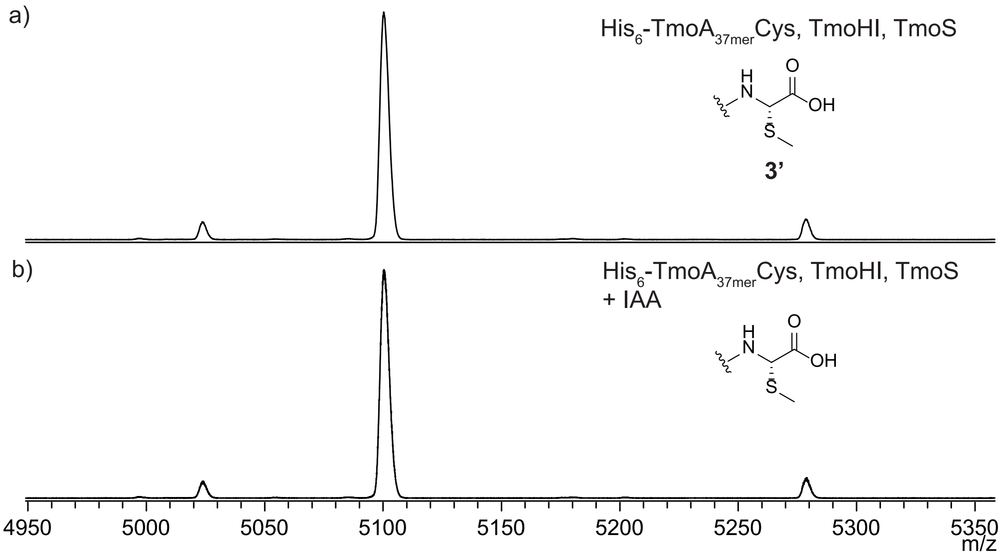

Figure S9. MALDI-TOF mass spectra showing that the C-terminal 37mer of TmoA is sufficient for enzymatic modifications by TmoHI and TmoS. a) The coexpression product of His_6-$TmoA_{37mer}Cys$, TmoHI and TmoS. Average m/z $[M+H]^+$ calculated for **3'**, the C-terminal 38mer of peptide **3**: 5101, observed 5100. b) Treatment of the co-expression product in panel a with IAA. Average m/z $[M+H]^+$ calculated: 5101, observed 5100. The lack of labeling by IAA suggests the identity of the co-expression product to be the C-terminal 38mer of peptide **3**.

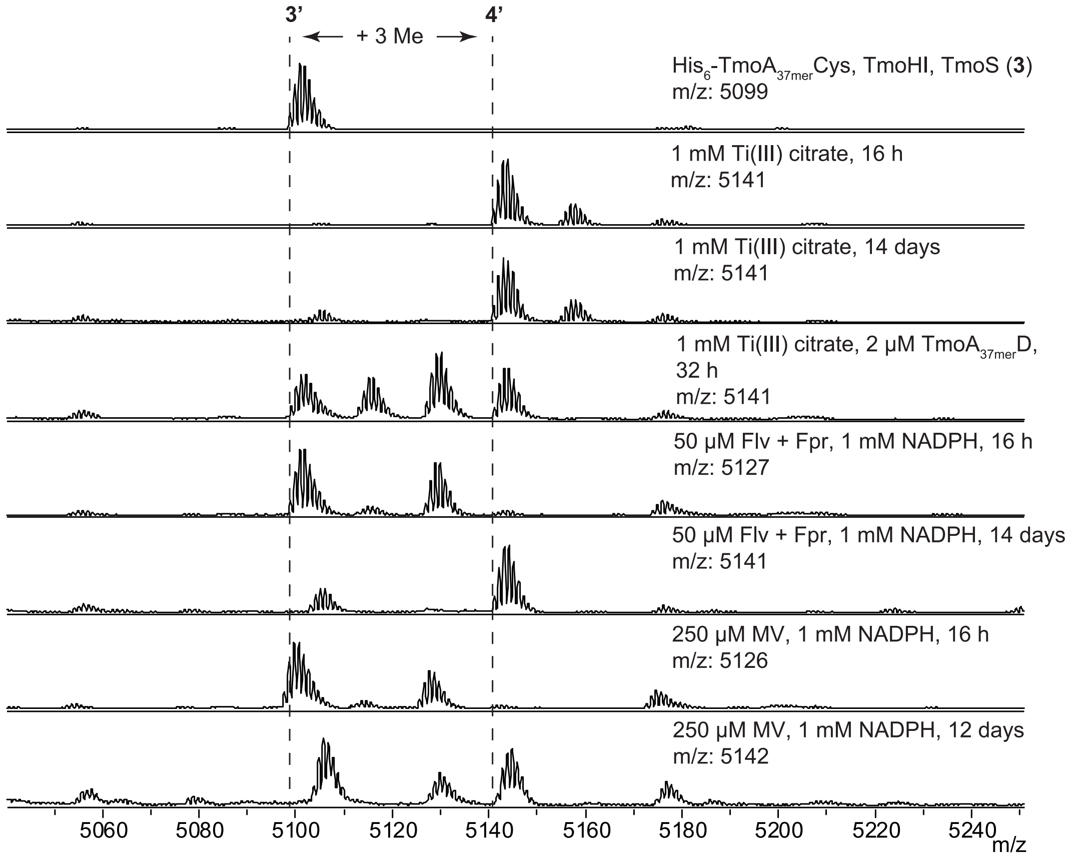

Figure S10. *In vitro* activity of TmoAD complex when different reductants were used. Flv: *E. coli* flavodoxin. Fpr: *E. coli* flavodoxin reductase. MV: methyl viologen. To better resolve different methylation states by MALDI-TOF MS, we shortened the length of the carrier peptide to the C-terminal 37 amino acids of TmoA (termed $TmoA_{37mer}$; Fig. S9). The N-terminally truncated analog of peptide **3** (**3'**) was similarly generated by co-expressing His_6-$TmoA_{37mer}$Cys, TmoHI and TmoS (top spectrum). All other spectra show the products after in vitro treatment with $TmoA_{37mer}$D and the reducing agents indicated. The $TmoA_{37mer}$D complex was prepared by co-expressing His_6-$TmoA_{37mer}$ and TmoD. Unless otherwise stated, all reactions were carried out anaerobically at 16 °C with 180 µM substrate, 10 µM $TmoA_{37mer}$D complex and 1 mM SAM. Calculated monoisotopic m/z of the substrate $[M+H]^+$: 5098, observed 5099. Calculated monoisotopic m/z of the trimethylated product $[M+H]^+$: 5140. The observed monoisotopic m/z for the product in each case is listed in each panel. Ti(III) citrate turned out to be the most effective reductant for TmoD in vitro. A small amount of tetramethylated peptide (m/z 5155 monoisotopic) is observed in the most active systems. A peak at m/z 5174 is observed in all reactions with intensity irrespective of the methylation activity. We do not know its identity.

a)

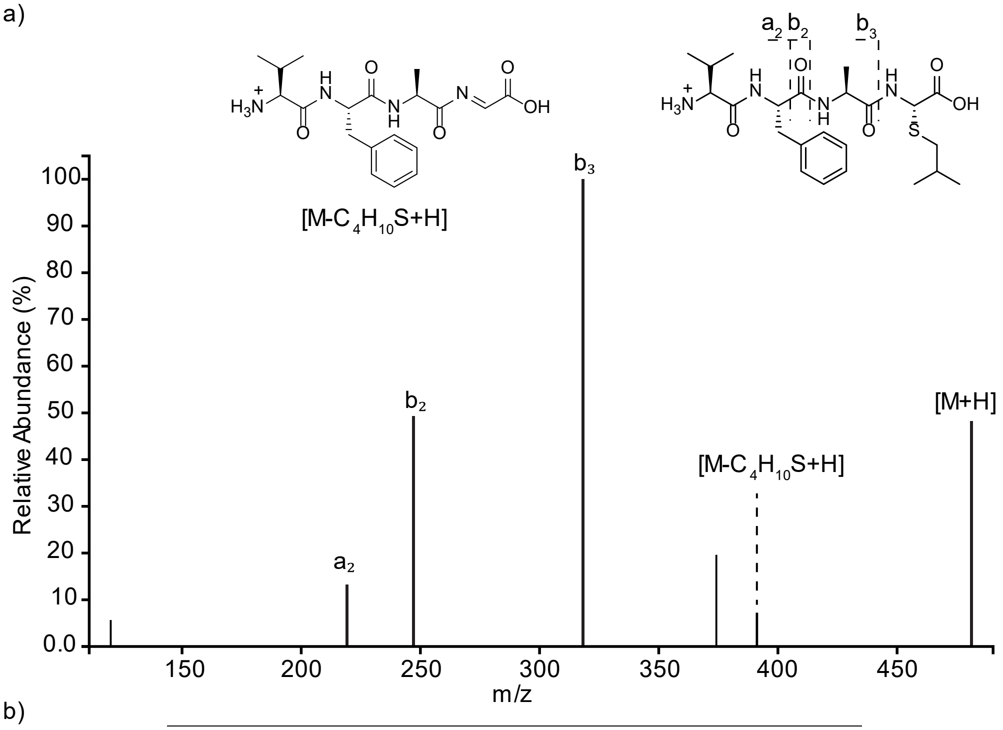

b)

Ion	Exp. Mass	Theo. Mass	Error (ppm)
a2	219.1489	219.1492	-1.3044
b2	247.1441	247.1441	-0.0019
b3	318.1810	318.1812	-0.6740
[M-C_4H_{10}S+H]	391.1965	391.1976	-2.8119
M+H	481.2480	481.2479	0.1680

Figure S11. TmoD trimethylated the C-terminal residue of peptide **3**. a) ESI-HRMS/MS of the C-terminal 4-mer peptide from the trypsin digest of peptide **4**. b) Theoretical and observed ions in the MS^2 spectrum.

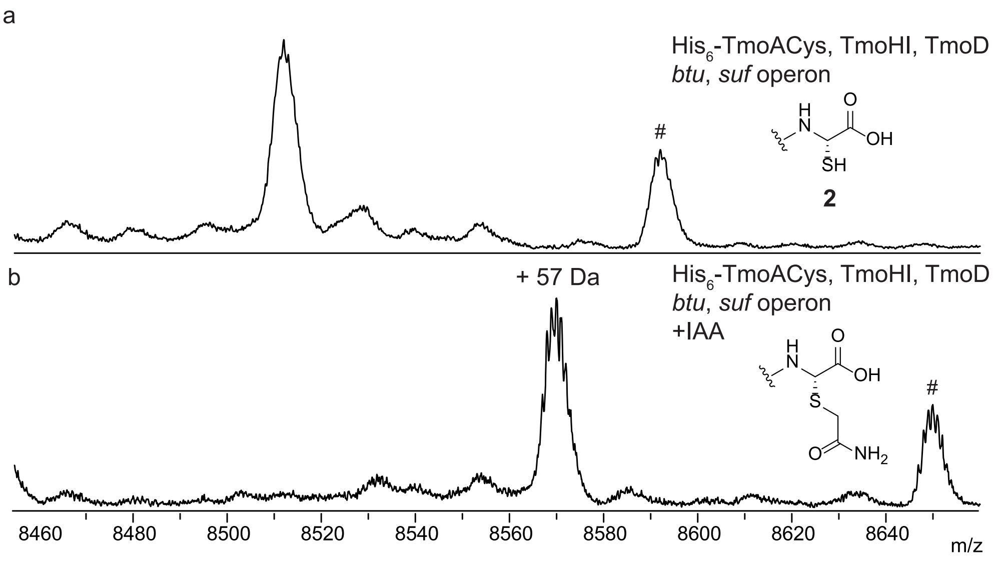

Figure S12. MALDI-TOF mass spectra showing that TmoD did not modify peptide **2**. a) TmoD did not install further modifications on TmoHI-modified TmoACys (**2**). Average m/z $[M+H]^+$ calculated: 8514, observed 8512. b) Treatment of the co-expression product in panel a with IAA. Average m/z $[M+H]^+$ calculated: 8571, observed 8570. This IAA labeling further supported the identity of the co-expression product to be peptide **2**. Peaks labeled with # indicate peptide with phosphorylation on the His-tag (8, 9) as shown by MS analysis after trypsin digest.

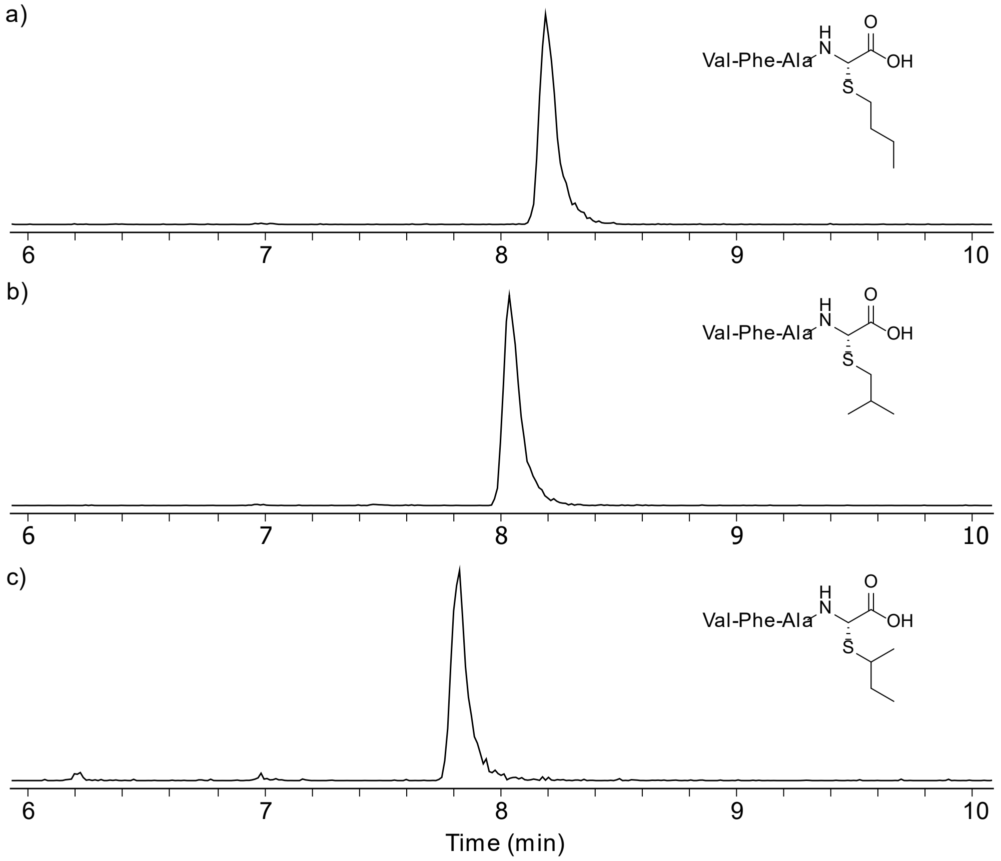

Figure S13. Extracted ion chromatograms (EICs, m/z 481.2479) of each of the VFAX peptides (X denoting the cysteine-derived amino acid). a) EIC of the *n*-butyl group containing standard. b) EIC of the isobutyl group containing standard. c) EIC of the *sec*-butyl group containing standard. The three standards have different retention times under the LC method two described in the Methods. The vertical axis corresponding to ion counts has been scaled for clarity.

A

B

C

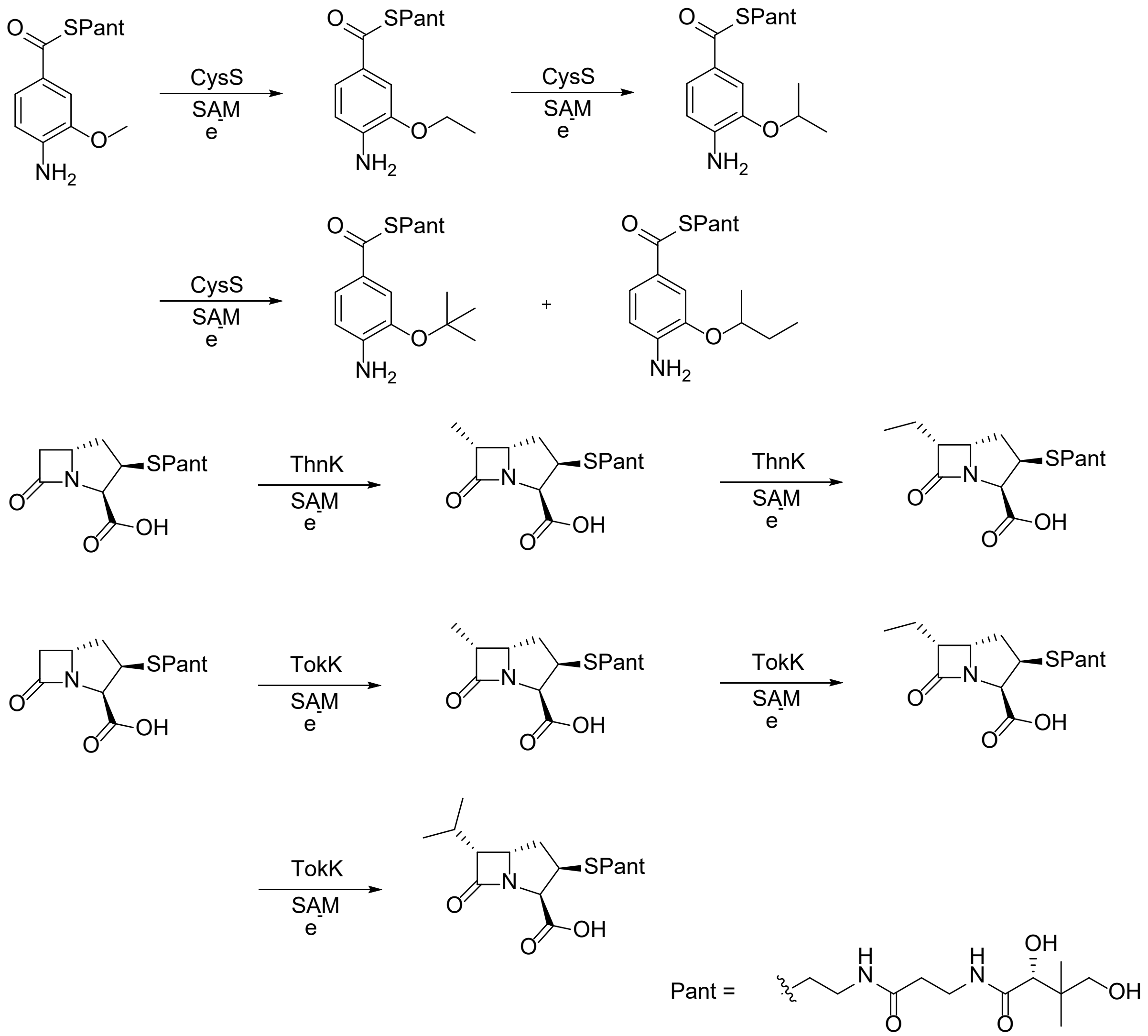

D

Percent Identity	TmoD	CysS	Thnk	TokK
TmoD				
CysS	24.5%			
Thnk	24.2%	29.8%		
TokK	25.3%	30.1%	79.4%	

Figure S14. Reactions catalyzed by cobalamin-dependent rSAM enzymes that methylate their substrates consecutively in vitro. (A) CysS methylates the methoxy group of its substrate to generate *tert*-butoxy and *sec*-butoxy groups. (B) ThnK installs the C6-ethyl chain of thienamycin. (C) TokK installs the C6-isopropyl chain of asparenomycin. (D) Comparison of sequence identity between TmoD, CysS, ThnK and TokK (10, 11). Although the reaction catalyzed by TmoD resembles the reaction catalyzed by TokK, the sequences of the two enzymes share low identity.

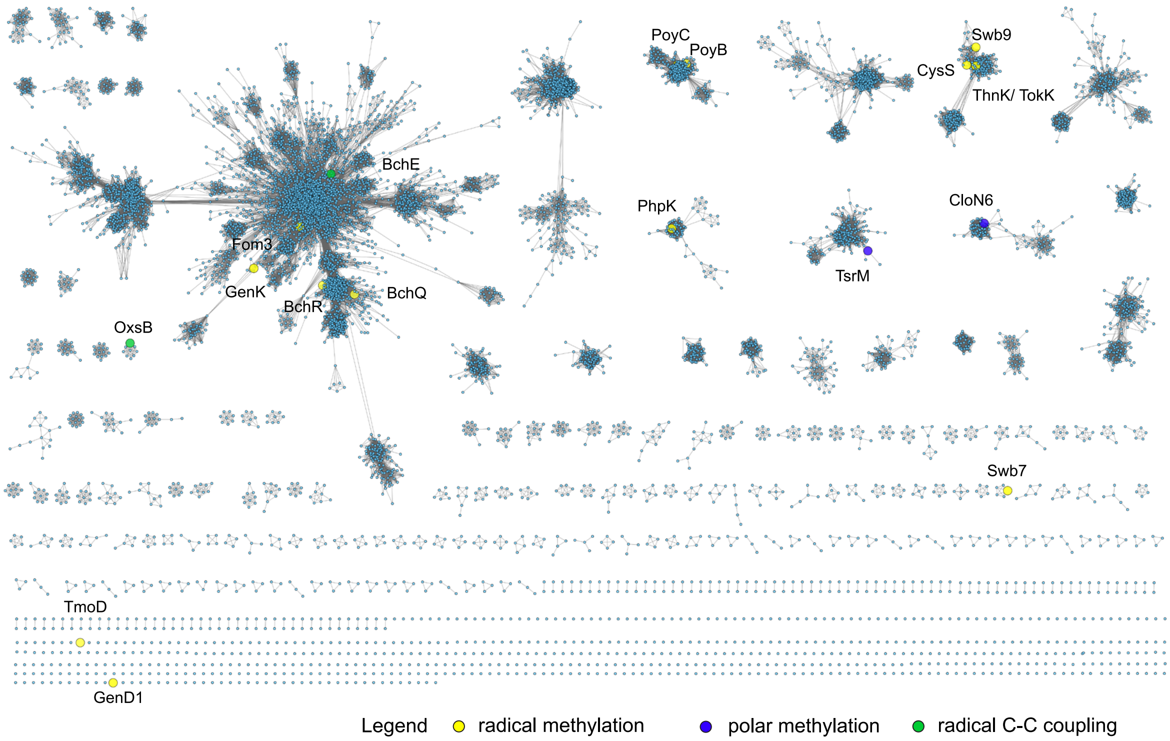

Figure S15. Sequence similarity network of cobalamin-dependent radical SAM enzymes (12). Representative enzymes are annotated and colored according to their reaction mechanisms (11). CysS, ThnK and TokK methylate their corresponding substrate iteratively in vitro and are in the same group. Swb9, PoyB, PoyC and BchQ have been proposed to also catalyze multiple methylation reactions but are in different groups. TmoD is currently a singleton. Given the diverse transformations catalyzed by this family of enzymes and the low coverage of characterized members in the network, prediction of enzymatic function based on sequence is at present challenging. Future characterization of this enzyme family will likely yield new insights into their functions and mechanisms.

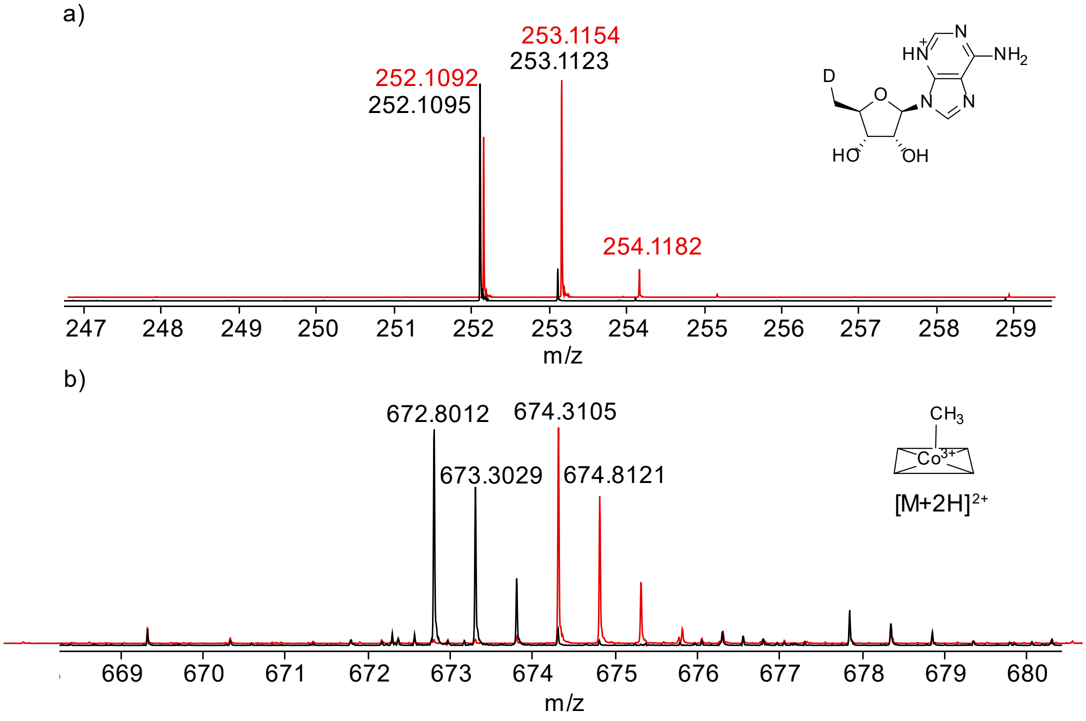

Figure S16. Overlaid ESI high-resolution mass spectra of 5'-deoxyadenosine (5'-dA) and methylcobalamin generated during the *in vitro* reaction of TmoD using labeled and unlabeled SAM. The reconstituted $TmoA_{37mer}D$ complex was used in the assay. Ions colored in red are from the reaction where d_3-SAM was used. a) Detection of 5'-dA. $[M+H]^+$ calculated 252.1091, observed 252.1095 (1.59 ppm error). $[d_1\text{-}M+H]^+$ calculated 253.1154, observed 253.1154 (0 ppm error). When d_3-SAM was used, approximately 58% of generated 5'-dA was deuterated, based on ion count integration. This result was consistent with the abstraction of one H and two D atoms during the trimethylation process. b) Detection of methylcobalamin. $[M+2H]^{2+}$ calculated 672.8012, observed 672.8012 (0 ppm error). $[d_3\text{-}M+2H]^{2+}$ calculated 674.3016, observed 674.3015 (-0.13 ppm error). The vertical axis corresponding to ion counts has been scaled for clarity.

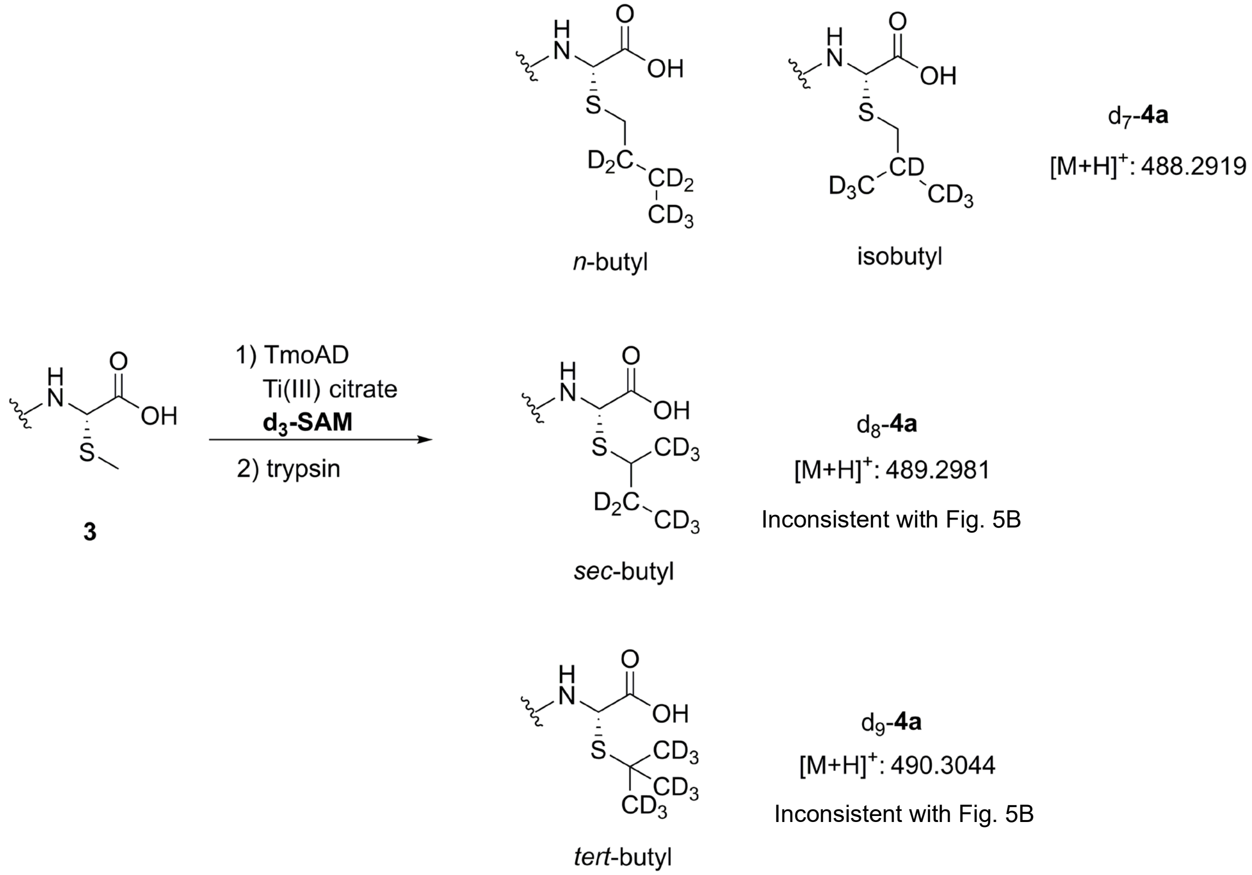

Figure S17. Deuterium labeling of the product peptide ruled out the *sec*-butyl or the *tert*-butyl group structure in the side chain of the last residue of peptide **4**. The C-terminal 4mer peptide of the product (denoted as peptide **4a**) incorporated seven deuterium atoms when d_3-SAM was used in the assay (Fig. 5B). Formation of different butyl groups by consecutive radical methylation using d_3-SAM will result in different degrees of deuterium atom incorporation in **4a** as shown in the figure. The experimental result in Fig. 5B is inconsistent with the formation of a *sec*-butyl group or a *tert*-butyl group at the C-terminal side chain. Therefore, the co-injection experiment and this deuterium labeling experiment collectively showed TmoD installed an isopropyl group on the methylthio group of peptide **3**.

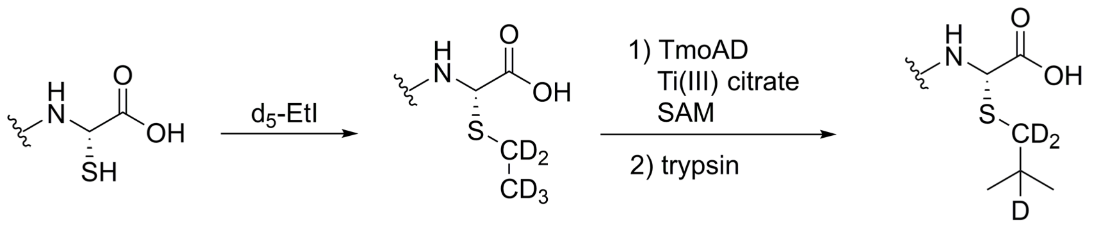

2 d5-**5** d3-**4a**

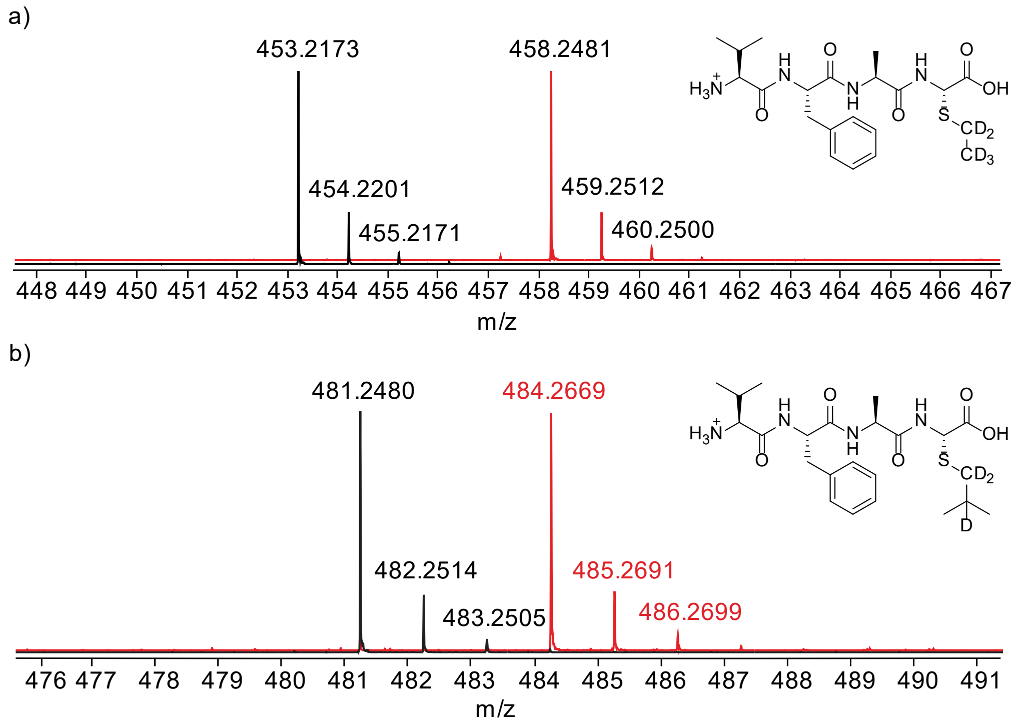

Figure S18. Overlaid ESI high-resolution mass spectra illustrating the transformation of peptide **5** to peptide **4a** during the *in vitro* reaction of TmoD. a) Detection of the unlabeled and labeled C-terminal 4-mer peptide from the trypsin digest of peptide **5**. [M+H]$^+$ calculated 453.2166; observed 453.2173 (-1.54 ppm error). [d$_5$-M+H]$^+$ calculated 458.2180; observed 453.2181 (0.22 ppm error). b) Detection of the unlabeled and labeled C-terminal 4-mer peptide (**4a**) from the trypsin digest of the product peptide **4**. [M+H]$^+$ calculated 481.2479; observed 481.2480 (-0.21 ppm error). [d$_3$-M+H]$^+$ calculated 484.2668; observed 484.2669 (-0.21 ppm error). Ions of product without deuterium label are shown in black and ions for product with deuterium labels are shown in red. The vertical axis corresponding to ion counts has been scaled for clarity.

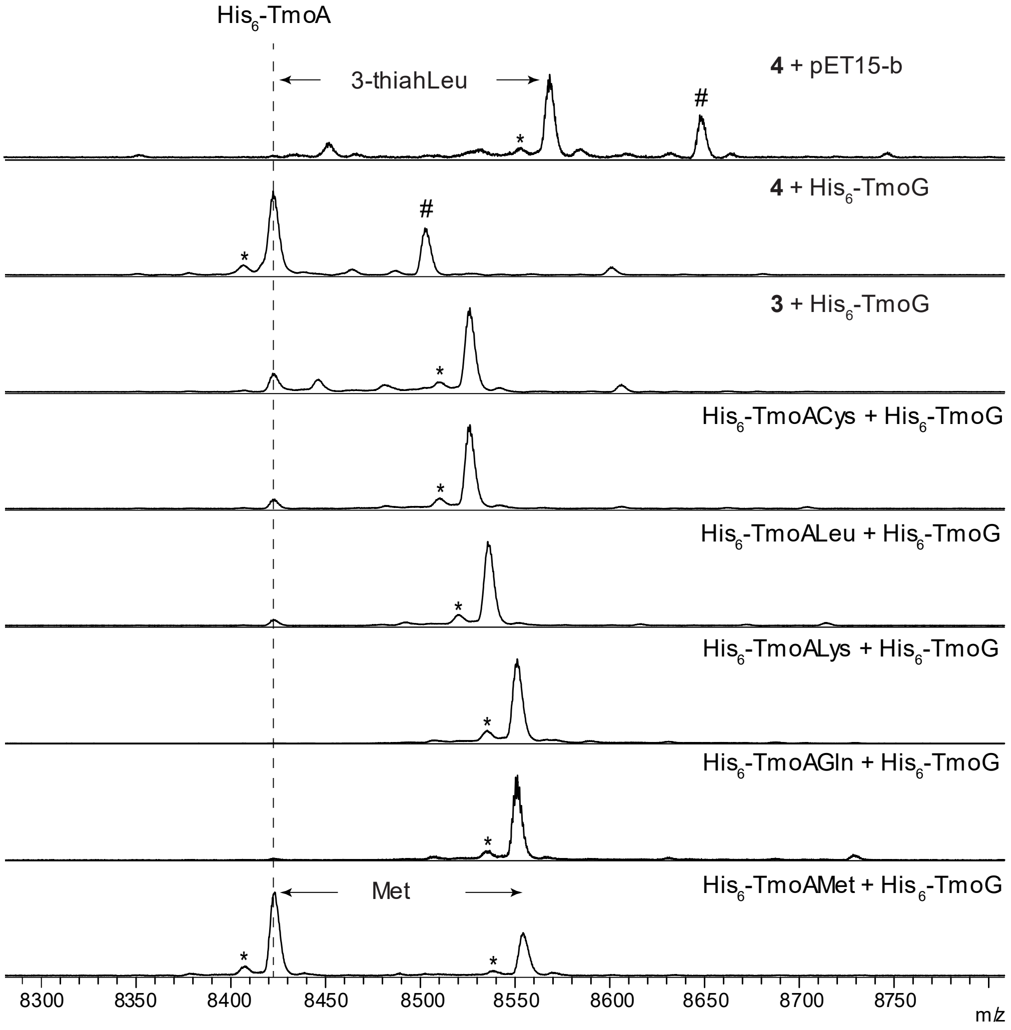

Figure S19. The substrate specificity of TmoG. Peptide **4** was treated with the lysate of *E. coli* containing an empty plasmid as control (top panel) to rule out endogenous *E. coli* proteases cleaving peptide **4**. Different peptides in the other panels were treated with the lysate of *E. coli* over-expressing His_6-TmoG. Dashed line indicates the conversion of those peptides to His_6-TmoA. Only peptide **4** and His_6-TmoAMet were significantly processed by His_6-TmoG to yield His_6-TmoA. Average m/z $[M+H]^+$ of His_6-TmoA calculated 8425; observed 8422 (second panel, from peptide **4**) and 8423 (last panel, from His_6-TmoAMet). Peaks labeled with # are likely peptide **4** or His_6-TmoA phosphorylated on the GSSHHHHHH His-tag sequence (8, 9). The results in the first two panels show such + 80 Da modification is not at the C-terminus of peptide **4**. Thus, this modification is unlikely to be installed by the biosynthetic enzymes in the *tmo* BGC.

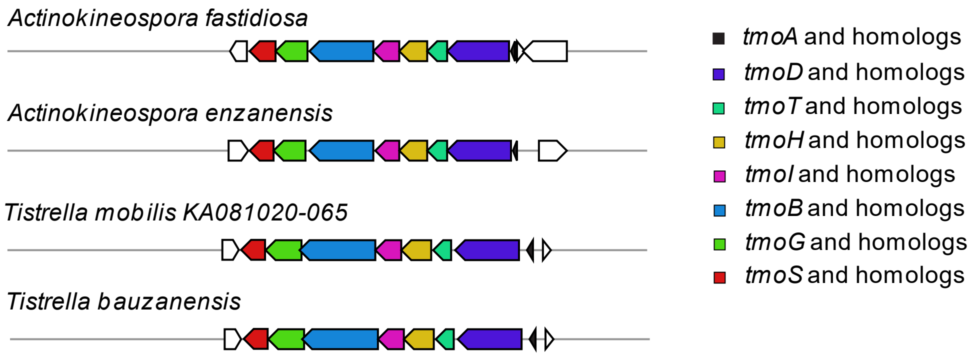

Figure S20. The *tmo* BGC and BGCs similar to it. The BGC investigated in this study is from *Tistrella mobilis* KA081020-065. Similar BGCs are found in the genus *Tistrella* and *Actinokineospora*.

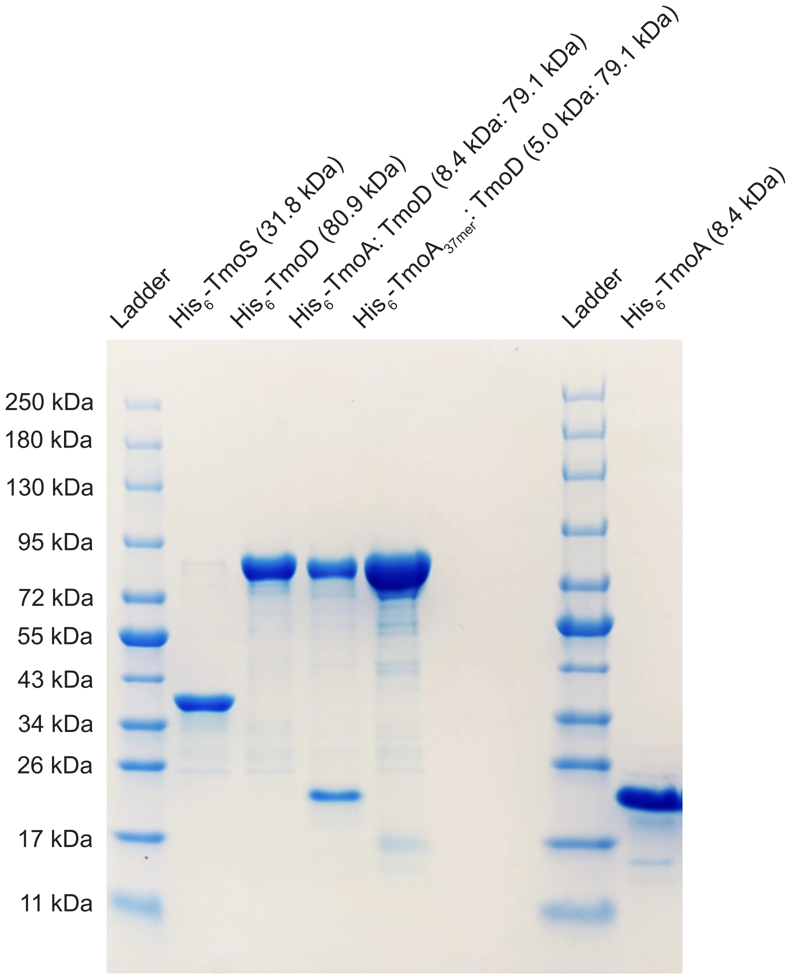

SDS-PAGE analysis of all proteins used in this study. The ladder used was Blue Prestained Protein Standard, Broad Range (11-250 kDa, NEB# P7718S). His$_6$-TmoA runs higher than expected but MS data show the peptide has the desired sequence.

Table S1. Primers used in this study

Name	Sequence
TmoAF	ATGACCGAACGCGATG
TmoAR	TTACGCAAACACCTTGCTTTC
TmoA_pRSF_F	GAGATTATCGAAAGCAAGGTGTTTGCGTAAAGCCAGGATCCGAATTCG
TmoA_pRSF_R	CGTAGCAGTCGCATCGCGTTCGGTCATGTGGTGATGATGGTGATGG
TmoB1F	GTATATTAGTTAAGTATAAGAAGGAGATATACATATGACCGTGGCGAC
TmoB1R	CAGTACTGGGGCCAG
TmoB2F	CTGCCGAGCCGGATC
TmoB2R	TTACGCGGGATCTTTAGTGG
TmoB_BBF	CAGCCCCCACTAAAGATCCCGCGTAAGCAGATCTCAATTGGATATCGG
TmoB_BBR	GTCGCCACGGTCATATGTATATCTCCTTCTTATACTTAACTAATATACTAAGATG
TmoACysF	TGCTAAAGCCAGGATCCGAATTC
TmoACysR	CGCAAACACCTTGCTTTC
TmoHI_F	AAGGAGATATACAATGGATAAACCGG
TmoHI_R	TTAGGCGCCAACCAC
TmoHI_BBF	CGAACGCGGTGCGGTGGTTGGCGCCTAAAGCCAGGATCCGAATTCG
TmoHI_BBR	GCGGTGCCGGTTTATCCATTGTATATCTCCTTTTAGCACGCAAACACCTTG
TmoSF	GGAGATATACATATGACTGCAGCACCTAC
TmoSR	TTAGCCAGGGGGAGTAATG
TmoS_BBF	GCAAGACCGCATTACTCCCCCTGGCTAAGCAGATCTCAATTGGATATCGG
TmoS_BBR	TTGTAGGTGCTGCAGTCATATGTATATCTCCTTCTTATACTTAACTAATATACTAAGATG
HisTmoS_F	ACTTCCAATCCACTGCAGCACCTACAACTG
HisTmoS_R	ACAGGTTCTCGTGGTGATGATGGTGATGGC
TmoD_F	GGAGATATACATATGACAGGTCACTTTCGTATGAG
TmoSD_g2F	CTATATGGCTCCAGATGGTAACAC
TmoSD_g1R	TCGTCCGTGTTACCATCTG
TmoD_R	TTAGGAACCGGTCGCC
TmoD_BBF	GTAGCGGAGGCGACCGGTTCCTAAGCAGATCTCAATTGGATATCGG
TmoD_BBR	CATACGAAAGTGACCTGTCATATGTATATCTCCTTCTTATACTTAACTAATATACTAAGATG
TmoACysHID_F	GATATACAATGACAGGTCACTTTCGTATGAGTCAAGATCCGGATCTGC
TmoACysHID_R	TCCTTTTAGGCGCCAACCACCGCACC
TmoA37mer_F	GACACGGCAACGGACGGC

TmoA37mer_R	GTGGTGATGATGGTGATGGCTG
pet15b_TmoG_F	CCGTCAGGGTCGTCATATGGCTGCCGCGC
pet15b_TmoG_R	GCATGATGTTAGAATTAATCCGCTAACTCGAGGATCCGGCTG
TmoG_pet15b_F	CAGCCGGATCCTCGAGTTAGCGGATTAATTCTAACATCATGCC
TmoG_pet15b_R	GTGCCGCGCGGCAGCCATATGACGACCCTGACGGC
TmoAMetF	ATGTAAAGCCAGGATCCGAATTC
TmoALysF	AAATAAAGCCAGGATCCGAATTC
TmoAGlnF	CAGTAAAGCCAGGATCCGAATTC
TmoALeuF	CTGTAAAGCCAGGATCCGAATTC
btu-pBADCDF_F	TACACCTTGTCTGGCAGCTACACCTTCTGAGTACCCGGGGATC CTC
btu-pBADCDF_R	TTGTCGCTGTTGTTGGCGGGCAAGTGTCAGCATGGTGAATTCC TCCTGC
pBAD1030_btuF	ATGCTGACACTTGCCC
pBAD1030_btuR	TCAGAAGGTGTAGCTGCC
HisTmoD_F	CATCACCACGAGAACCTGTACTTCCAATCCACAGGTCACTTTC GTATGAGTC
HisTmoD_R	GAGCTCGAATTCGGATCCTGGCTTTAGGAACCGGTCGC
HisTmoD_BBF	CGGAGGCGACCGGTTCCTAAAGCCAGGATCCGAATTCG
HisTmoD_BBR	GGATTGGAAGTACAGGTTCTCGTGGTGATGATGGTGATGG

References:

1. McLaughlin MI & van der Donk WA (2018) Stereospecific radical-mediated B_{12}-dependent methyl transfer by the fosfomycin biosynthesis enzyme Fom3. *Biochemistry* 57(33):4967-4971.
2. Chung CT, Niemela SL, & Miller RH (1989) One-step preparation of competent *Escherichia coli*: transformation and storage of bacterial cells in the same solution. *Proc. Natl. Acad. Sci. U. S. A.* 86(7):2172-2175.
3. Hänzelmann P*, et al.* (2004) Characterization of MOCS1A, an oxygen-sensitive iron-sulfur protein involved in human molybdenum cofactor biosynthesis. *J. Biol. Chem.* 279(33):34721-34732.
4. Nishihara K, Kanemori M, Kitagawa M, Yanagi H, & Yura T (1998) Chaperone coexpression plasmids: differential and synergistic roles of DnaK-DnaJ-GrpE and GroEL-GroES in assisting folding of an allergen of Japanese cedar pollen, Cryj2, in Escherichia coli. *Appl. Environ. Microbiol.* 64(5):1694-1699.
5. Zhang Z, Mahanta N, Hudson GA, Mitchell DA, & van der Donk WA (2017) Mechanism of a class C radical S-adenosyl-l-methionine thiazole methyl transferase. *J. Am. Chem. Soc.* 139(51):18623-18631.
6. Lanz ND*, et al.* (2012) RlmN and AtsB as models for the overproduction and characterization of radical SAM proteins. *Methods Enzymol.* 516:125-152.
7. Brademan DR, Riley NM, Kwiecien NW, & Coon JJ (2019) Interactive peptide spectral annotator: A versatile web-based tool for proteomic applications*. *Mol. Cell. Proteom.* 18(8, Supplement 1):S193-S201.
8. Du P*, et al.* (2005) Phosphorylation of serine residues in histidine-tag sequences attached to recombinant protein kinases: a cause of heterogeneity in mass and complications in function. *Protein. Expr. Purif.* 44(2):121-129.
9. Singh H, Verma D, & Bardiaux B (2019) Single-site phosphorylation within the His-tag sequence attached to a recombinant protein. *Anal. Biochem.* 570:62-64.
10. Altschul SF, Gish W, Miller W, Myers EW, & Lipman DJ (1990) Basic local alignment search tool. *J. Mol. Biol.* 215(3):403-410.
11. Knox HL, Sinner EK, Townsend CA, Boal AK, & Booker SJ (2022) Structure of a B(12)-dependent radical SAM enzyme in carbapenem biosynthesis. *Nature* 602(7896):343-348.
12. Bridwell-Rabb J, Li B, & Drennan CL (2022) Cobalamin-dependent radical S-adenosylmethionine enzymes: capitalizing on old motifs for new functions. *ACS Bio. Med. Chem. Au*. doi.org/10.1021/acsbiomedchemau.1c00051.
